# High-throughput quantification of more than 100 primary- and secondary-metabolites, and phytohormones by a single solid-phase extraction based sample preparation with analysis by UHPLC–HESI–MS/MS

**DOI:** 10.1186/s13007-016-0130-x

**Published:** 2016-05-26

**Authors:** Martin Schäfer, Christoph Brütting, Ian T. Baldwin, Mario Kallenbach

**Affiliations:** Department of Molecular Ecology, Max-Planck-Institute for Chemical Ecology, Hans-Knöll-Str. 8, 07745 Jena, Germany

**Keywords:** Phytohormones, Jasmonate, Salicylic acid, Abscisic acid, Gibberellin, Auxin, Cytokinin, Secondary metabolites, Primary metabolites, Solid-phase extraction

## Abstract

**Background:**

Plant metabolites are commonly functionally classified, as defense- or growth-related phytohormones, primary and specialized metabolites, and so forth. Analytical procedures for the quantifications of these metabolites are challenging because the metabolites can vary over several orders of magnitude in concentrations in the same tissues and have very different chemical characteristics. Plants clearly adjust their metabolism to respond to their prevailing circumstances in very sophisticated ways that blur the boundaries among these functional or chemically defined classifications. But if plant biologists want to better understand the processes that are important for a plant’s adaptation to its environment, procedures are needed that can provide simultaneous quantifications of the large range of metabolites that have the potential to play central roles in these adjustments in a cost and time effective way and with a low sample consumption.

**Results:**

Here we present a method that combines well-established methods for the targeted analysis of phytohormones, including jasmonates, salicylic acid, abscisic acid, gibberellins, auxins and cytokinins, and extends it to the analysis of inducible and constitutive defense compounds, as well as the primary metabolites involved in the biosynthesis of specialized metabolites and responsible for nutritional quality (e.g., sugars and amino acids). The method is based on a single extraction of 10–100 mg of tissue and allows a broad quantitative screening of metabolites optimized by their chemical characteristics and concentrations, thereby providing a high throughput analysis unbiased by the putative functional attributes of the metabolites. The tissues of *Nicotiana attenuata* which accumulate high levels of nicotine and diterpene glycosides, provide a challenging matrix that thwarts quantitative analysis; the analysis of various tissues of this plant are used to illustrate the robustness of the procedure.

**Conclusions:**

The method described has the potential to unravel various, until now overlooked interactions among different sectors of plant metabolism in a high throughput manner. Additionally, the method could be particularly beneficial as screening method in forward genetic approaches, as well as for the investigation of plants from natural populations that likely differ in metabolic traits.

**Electronic supplementary material:**

The online version of this article (doi:10.1186/s13007-016-0130-x) contains supplementary material, which is available to authorized users.

## Background

The continuous advances in the resolution and speed of chromatography and mass spectrometry has brought plant biologists to the privileged position that many laboratories now have direct access to instrumentation capable of quantifying the vast majority of physiologically and ecologically relevant plant compounds. However, many methods used with this advanced instrumentation still suffer from two challenges that limit their full power from being realized: (1) the analytical challenge of the vast differences in abundance and chemical properties of functionally related compounds that confound their simultaneous analysis and (2) the conceptual challenge of the tradition of grouping of compounds into simplified compound clades (e.g., “growth hormones”, “defense hormones”, “nutritive substance”) that might not be wrong *per se*, but often only cover a small portion of their functional characteristics. Plant metabolism is known to be highly dynamic and interconnected, and it will be important for plant biologists to overcome these analytical and conceptual limitations to understand the processes that mediate a plant’s adaptation to its environment.

Plants reorganize their metabolism as they establish vegetative structures, acquire nutrients, produce viable offspring, survive drought and other abiotic stresses, as well as navigate the challenges of maintaining mutualistic relationships while thwarting the advances of parasitic or herbivorous organisms. Phytohormones are important regulators of plant growth and development, as well as for the adaptation to their respective environment. Often, phytohormones are classified by their most prominent function, such as ‘growth hormones’ (gibberellins, GAs; auxins, AXs and cytokinins, CKs) or ‘defense hormones’ (jasmonic acid, JA and salicylic acid, SA). Many available protocols for phytohormone analysis retain these artificial constructs by concentrating either on one or another functional group. However, these hormones have also been shown to participate in adaptations different from their classical function. JAs, for example, are well known to regulate flower development [12, 39] and senescence induction [42] and cytokinins also participate in interactions with pathogens and herbivores [8, 38]. Additionally, many phytohormone pathways are known to interact with each other. This interaction can occur for instance at the signaling level, as shown for the GA pathway that can amplify the JA signaling by the binding of the GA-regulated DELLA proteins to the JASMONATE ZIM-DOMAIN (JAZ)-suppressors [17], the negative regulators of JA signaling. Phytohormones can also influence each other at a metabolic level, such as reported for AXs that can reduce the CK levels by promoting the cytokinin oxidase/reductase (CKX)-mediated degradation of CKs [6, 47]. Another limitation of many studies is that often hormone regulation studies focus either on specific secondary or primary metabolites, but rarely on multiple sectors of the plant metabolism. However, phytohormone pathways are also known to interact on these levels. CKs, for example, can regulate phenylpropanoid [4, 16] and polyamine levels [7, 44], which are precursors of JA-inducible defense compounds, such as caffeoylputrescine [13]. Phytohormones can also affect the nutritional value, as well as the abundance of defense compounds simultaneously. The associated effects on other organisms might be complementary, neutral or even antagonistic thereby complicating the analysis of plant interactions with other organisms (e.g., pathogens, herbivores or mutualists). For example, in a previous study, we observed that higher CK levels increased the leaf damage inflicted by the mirid bug *Tupiocoris notatus* on *Nicotiana attenuata* plants [33]. CKs were shown to amplify herbivory-induced defense responses in *N. attenuata* [34], but they are also known to increase concentrations of primary metabolites [20, 32] that might positively affect herbivore performance [21, 28] and therefore probably compensate for potential changes in plant defense. Unfortunately, most of these proposed effects still remain to be confirmed for specific plant-herbivore interactions.

Primary metabolites are not only important targets for phytophagous organisms, but also serve diverse functions that span the interface between primary and secondary metabolism. Amino acids are the building blocks for protein biosynthesis, but also serve as precursors for various secondary metabolites, such as the phenylpropanoid pathway derived coumarins, flavonoids and anthocyanins [43], as well as glucosinolates [15]. Additionally, they contribute to the formation of phytohormones, like indole-3-acetic acid (IAA; [27]), SA [9] or the bioactive JA-isoleucine conjugate (JA-Ile; [46]). Similarly, sugars are not only a basic unit of energy storage, but they can also act as signaling molecules [37] and the glycosylation of various phytohormones and secondary metabolites plays an essential role for the regulation of their activity, stability and localization [2, 29, 45].

Analytical methods that provide a broad overview about the various phytohormones, as well as primary and secondary metabolites would be highly beneficial for an understanding of the underlying metabolic adaptations that plants have evolved towards ecological stresses. The simultaneous analysis of many compounds reduces the amount of plant material required, the sample preparation time and the use of consumables, which reduces the price per sample.

One analytical challenge for the simultaneous analysis of multiple plant metabolites are their different abundances. While 1 g leaf tissue can contain µmol amounts of specific amino acids and some secondary metabolites, phytohormones might be present and functioning in the fmol range. Therefore it is necessary to group the compounds that are suitable for a simultaneous analysis and optimize the sample preparation, accordingly. The analysis of low abundant compounds, for example, needs additional enrichment, but also purification steps to prevent signal suppression and possible column overload due to the sample matrix, while other compounds require a dilution before analysis. Additionally, it is important to prevent enzymatic activity throughout the extraction procedure and to separate compounds that might be converted into each other. Also for the later chromatographic separation a grouping into substances with similar requirements can be helpful. Kojima et al. [23] presented a high throughput extraction and purification procedure for phytohormones that can be a suitable basis for such a screening method. The method uses an extraction in an acidified MeOH–water buffer at low temperatures similar to the method described by Bieleski [5] (without chloroform to prevent the extensive extraction of lipids) and a subsequent purification by a two-step solid-phase-extraction (SPE) as described by Dobrev and Kamínek [11]. After a cleanup by a reverse-phase (RP) column, the separation is done by a mixed RP and cation-exchange column (Oasis MCX), which allows for the separation of cationic CK-bases, ribosides and glucosides, from anionic auxin, gibberellins and abscisic acid (ABA), as well as CK-nucleotides [11, 23]. The reduced sample complexity could also aid in the analysis of other low abundant compounds. And indeed the same column (Oasis MCX) was described in another protocol to be suitable for the purification of other phytohormones, such as JAs and SA [3]. A combined approach was used already e.g., by Djilianov et al. [10], Záveská Drábková et al. [48] and Zhang et al. [49].

Additional compounds that are of interest for biochemical and ecological studies are amino acids and sugars. For amino acids it was shown by Jander et al. [19] that the extraction in an acidified ethanol–water buffer and the subsequent analysis by liquid chromatography coupled to tandem mass spectrometry (LC–MS/MS) represents a time-efficient and reliable method. Also for the analysis of sugars, analytical methods are available. But, to prevent the high costs associated with many enzymatic assays or the additional derivatization steps, which are required for a gas chromatography-based analysis [25], it seems most suitable for a screening method to utilize a MS based method relying on the separation by hydrophilic interaction liquid chromatography (HILIC) [18, 26]. Secondary metabolites are often species-specific and their analysis has to be adjusted accordingly to the plant taxa. However, they often belong to similar compound classes and their analysis might therefore have related requirements. For the method described here, we choose as examples, caffeoylputrescine and nicotine, as well as scopoletin, chlorogenic acid and rutin, representing an inducible and a constitutive (partially inducible) defense compound against herbivores, a phytoalexin (de novo produced antimicrobial compound), phytoanticipin (pre-formed antimicrobial compound), as well as a compound assumed to play a role in UV-protection, respectively. Chemically, these examples represent phenolamides, alkaloids, depsides and flavonol glycosides. Additionally, important precursors were included to provide a broad overview of the metabolic changes within a plant. With scopolamine we include another prominent plant defense that can be found in different genera of the family *Solanaceae* [14].

The investigation by Balcke et al. [3] demonstrated that a close analog to the Oasis MCX column, the Chromabond HR-XC column, provides similar chromatographic properties but are less costly. Additionally, the column material is reported to be robust even under extreme pH- and solvent-conditions—raising the question if also a cleanup procedure could be applied, enabling the re-use of these columns, and lowering the per sample costs of the analysis further.

The presented method describes an extraction, purification and analysis method that enables a broad overview about levels of various growth and defense related phytohormones, primary metabolites, as well as secondary metabolites that play a role in plant interactions with their environment. The method allows for the analysis of more than 100 compounds in one extraction, is doable roughly in 6 days (for 96 samples) including all extraction and purification steps (~1 day) as well as the MS/MS based analysis (~5 days).

## Results and discussion

### UHPLC–HESI–MS/MS

For the analysis we used an Ultra High Performance Liquid Chromatography (UHPLC) coupled to a triple quad mass spectrometer equipped with a heated electrospray ionization (HESI) source. First, for all compounds of interest, labeled and/or unlabeled standards were used in direct injections to determine the m/z values for the precursor ions, the MS/MS fragmentation patterns and to optimize the fragmentation conditions (Additional file 1: Tables S1–S8). For compounds measured without isotopically labeled internal standard we included additional MS/MS traces as *Qualifiers* for the verification of compound identity, if possible.

The compounds were divided in 7 groups and suitable UHPLC methods were developed based on their behavior during the sample preparation, chromatographic characteristics and abundance. For the chromatographic separation of most compounds, including all phytohormones, amino acids and phenylpropanoids (Methods 1A, 1B, 2A, 2B and 3) we used a Zorbax Eclipse XDB-C18 column with acidified water and MeOH as the mobile phase in gradient mode. For the separation of the alkaloids (Method 1C), we used a Gemini C18 column under alkaline conditions to prevent the protonation of the analytes which improved their separation with reversed phase chromatography. For separations of the sugars (Method 1D), we used an acetonitrile–water gradient on an apHera amino (NH_2_) column (HILIC) that is optimized for saccharide separations. The gradients used for each UHPLC method are given in Additional file 1: Tables S9–S15; these were optimized to prevent co-elution of analytes with similar multi-reaction-monitoring (MRM) settings, to reduce matrix effects, and to be sufficiently short for high-throughput analysis of large sample sets. Each run includes a cleaning and reconditioning segment to help maintain the chromatographic separations of the column throughout the analysis of related sample sets. Standards were used to identify the retention times (RT) of the analytes for each method.

For the few compounds for which no standards were accessible, MRM settings were defined based on published MRM conditions and the RT’s were identified by injecting plant extracts with known elevated concentrations of the respective compounds (indicated in Additional file 1: Tables S2–S8). Additionally, the relative chromatographic behavior compared to known standard substances was used to confirm these inferences.

The MRM settings and RTs are summarized in Additional file 1: Tables S2–S8, the source settings in Additional file 1: Table S1 and the chromatographic conditions are summarized in Additional file 1: Tables S9–15.

For quantification, we used various deuterated phytohormone standards, a mix of ^13^C, ^15^N-labeled amino acids from a commercially available algae extract, sorbitol and 4-methylumbelliferone (4-MU). In cases where identical isotopically labeled standards were not available, we quantified these compounds using a simultaneously measured standard and a respective response factor. The internal standards for quantification and response factors (if applicable) are summarized in Additional file 1: Tables S2–S8. The standards for phytohormones and other low abundance compounds were added to the extraction buffer (for Methods 2A, 2B and 3). The standards for high abundance compounds, such as amino acids and sugars were added during the dilution step, to reduce the consumption of standards. Additionally, these high-abundance standards might otherwise accumulate in the other, more concentrated Fractions (2A, 2B and 3) and suppress ionization of other analytes.

**Fig. 1:**
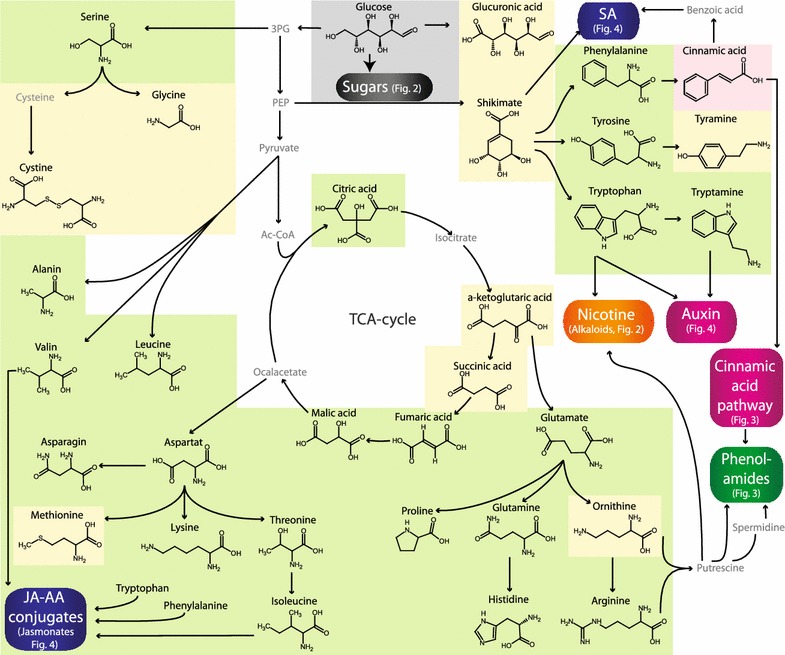
Overview of metabolites analyzed by the presented procedure: Part I. The *background color* indicates the specific MS method they are part of (Methods 1A *green,* 1B *yellow,* 1C *orange,* 1D *grey,* 2A *blue,* 2B *pink*, 3 *light blue*). Other metabolites are presented in more detail in Figs. 2, 3 and 4. Compounds that were not included in the analysis are only given by name and depicted in *grey front color*. *JA–AA conjugates* jasmonic acid–amino acid conjugates, *SA* salicylic acid

**Fig. 2:**
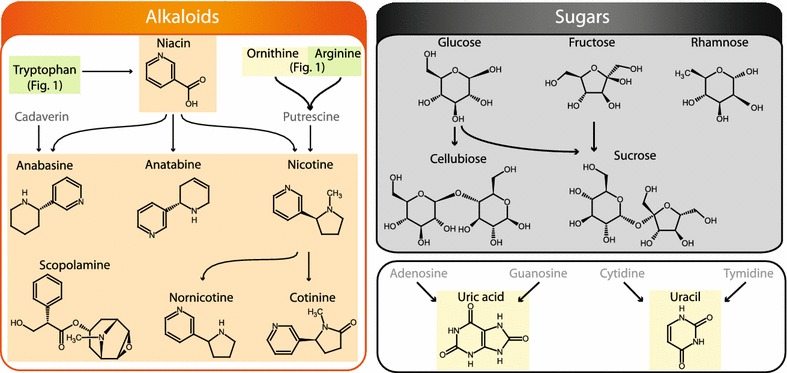
Overview of metabolites analyzed by the presented procedure: Part II. The *background color* indicates the specific MS method they are part of (Methods 1A *green,* 1B *yellow,* 1C *orange*, 1D *grey*, 2A *blue,* 2B *pink,* 3 *light blue*). Other metabolites are presented in more detail in Figs. 1, 3 and 4. Compounds that were not included in the analysis are only given by name and depicted in *grey front color*

**Fig. 3:**
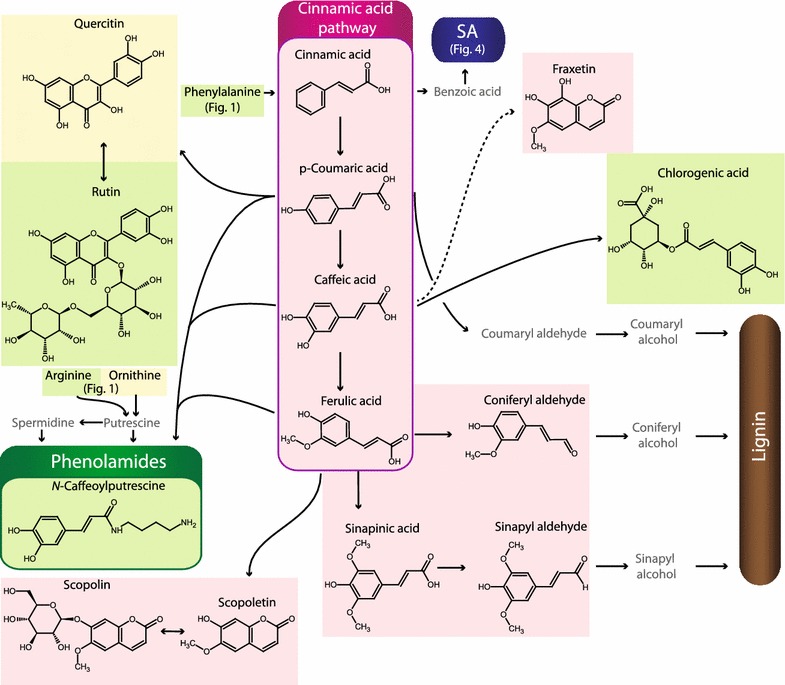
Overview of metabolites analyzed by the presented procedure: Part III. The *background color* indicates the specific MS method they are part of (Methods 1A *green,* 1B *yellow,* 1C *orange*, 1D *grey,* 2A *blue,* 2B *pink,* 3 *light blue*). Other metabolites are presented in more detail in Figs. 1, 2 and 4. Compounds that were not included in the analysis are only given by name and depicted in *grey front color*. *SA* salicylic acid

**Fig. 4:**
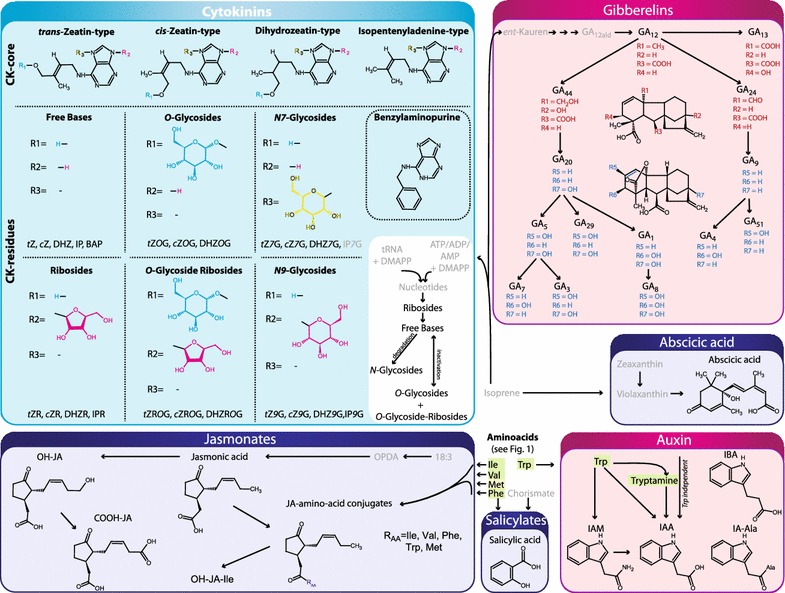
Overview of metabolites analyzed by the presented procedure: Part IV. The *background color* indicates the specific MS method they are part of (Methods 1A *green,* 1B *yellow,* 1C *orange,* 1D *grey,* 2A *blue,* 2B *pink,* 3 *light blue*). Other metabolites are presented in more detail in Figs. 1, 2 and 3. Compounds that were not included in the analysis are only given by name and depicted in *grey front color*. 18:3, α-linolenic acid; *c*Z, *cis*-zeatin; DHZ, dihydrozeatin; GA_n_, gibberellin An; IAA, indole-3-acetic acid; IAM, indole-3-acetamide; IA-Ala, indole-3-acetyl-alanine; IBA, indole-3-butyric acid; IP, isopentenyladenine; JA, jasmonic acid; OPDA, 12-oxo-phytodienoic acid; *t*Z, *trans*-zeatin

Figures 1, 2, 3 and 4 give an overview about the compounds that were measured with the described analytical procedure and indicates the specific UHPLC–HESI–MS/MS methods used for each analyte.

### Extraction and purification

**Fig. 5:**
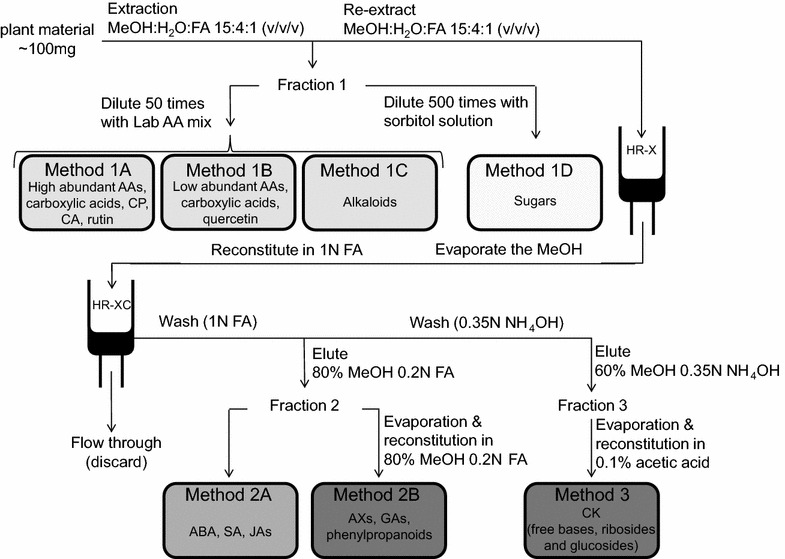
Overview about the extraction and purification protocol. Samples are extracted with acidified MeOH (containing isotope labeled phytohormone standards and 4-methylumbelliferone). An aliquot is used as Fraction 1 for the analysis of amino acids, various carboxylic acids, high abundance 2nd metabolites (e.g., caffeoylputrescine, chlorogenic acid, nicotine and rutin) and sugars. The samples were diluted with aqueous solutions containing either ^13^C, ^15^N-labeled amino acids or sorbitol, as internal standards, before the analysis. The remaining extract was combined with the re-extract of the pellet and purified on two solid-phase extraction (SPE) columns (HR-X and HR-XC). Analytes were retained on the second HR-XC column until sequential elution. Fraction 2 was used for the analysis of acidic phytohormones (ABA, SA, AXs and JAs), as well as for various compounds of the phenylpropanoid pathway. The low abundance compounds from Fraction 2 were measured after an additional concentration step. The Fraction 3 (CKs) was also concentrated before analysis. *AAs* amino acids, *ABA* abscisic acid, *AXs* auxins, *CA* chlorogenic acid, *CKs* cytokinins, *CP* caffeoylputrescine, *FA* formic acid, *GAs* gibberellins, *HR-X* and *HR-XC* solid-phase extraction columns, *JAs* jasmonates, *Lab AA mix* algae extract containing ^13^C, ^15^N-labeled amino acids, *SA* salicylic acid

Figure 5 gives an overview of the extraction and purification protocol used. For extraction and purification of low abundance phytohormones, we followed the protocol described by Dobrev and Kamínek [11] and Kojima et al. [23] with minor modifications; after extraction with acidified MeOH, we separated acidic phytohormones, such as the JAs, ABA, AXs and SA from the alkaline CK-ribosides, CK-glucosides and free bases on a mixed-mode RP-cation exchange SPE column (HR-XC). Importantly, the CK-phosphates were eluted separately from the other CK metabolites to prevent their conversion into other CK metabolites. For time- and cost-efficiency reasons, the CK-phosphates were not analyzed in the described method. Kojima et al. [23] presented a procedure for the dephosphorylation by alkaline phosphatase and a cleanup on another reversed phase SPE plate (Oasis HLB); these additional steps could be readily incorporated into the described procedure. The acidic phytohormones were analyzed by two separate methods due to their different natural abundances and ionizabilities in MS based analyses. While ABA, SA and JAs were directly measured in an aliquot of Fraction 2, for the analysis of AXs and GAs, fraction 2- was 20-fold concentrated by evaporation and reconstitution in a smaller amount of buffer.

To analyze the high abundant compounds, such as amino acids, sugars and nicotine, we used a diluted aliquot from the first extraction step (Fraction 1) without further clean up. To remain within the linear range, the samples were diluted 50–500 times for Fraction 1A/1B/1C and 1D, respectively (exceptions mentioned under "Methods"). Other less abundant metabolites, such as the hydroxycinnamic acids and related compounds from the phenylpropanoid pathway were analyzed together with AXs and GAs in the concentrated Fraction 2.

To apply the method for tissues with considerable different compound levels it might be necessary to adjust the injection amount, the dilution factor or concentration factor of the methods. In cases where concentrations of target compounds or matrix effects are unknown and a distribution of analytes into groups is not possible, a preliminary screening using dilution/concentration series of fractions from representative samples might be performed. First, all target compounds should be combined in one method per LC-column and -solvent system and sequentially distributed again into different methods based on the obtained results. Compounds from Method 2A, 2B or 3 that are too abundant can be shifted to Method 1A or 1B without additional problems. In contrast, shifting to a method for less abundant compounds requires additional investigations of the behavior of the compounds during the additional sample preparation steps, e.g., the retention and elution from the SPE columns and stability under the respective temperature- and pH-conditions. Acidic and neutral compounds should most likely accumulate in fraction 2, whereas alkaline compounds should elute from the HR-XC column in fraction 3 or the previous washing step. If a shift between the available methods isn’t sufficient and for compounds that rely on another column than the Zorbax Eclipse XDB-C18 (e.g., sugars and nicotine) it might be necessary to establish additional methods.

### Method validation

For method evaluation, we determined the linear range, the limit of detection (LOD) and limit of quantification (LOQ) of the instrument (LOD_i_ and LOQ_i_, respectively), the recovery rate for the purification and concentration procedure, as well as the matrix effect for a herbivory-induced leaf matrix. Additionally, we calculated the LOQ for the method (LOQ_m_; minimal amount per sample).

The LOD_i_ for most amino acids and compounds related to the phenylpropanoid pathway was in the low fmol range (usually below 20 fmol), while most small carboxylic acids (e.g., citric acid, fumaric acid, etc.) and all sugars ranged between 50 and 100 fmol. Exceptions were e.g., Gly with a LOD_i_ of nearly 600 fmol, as well as cinnamic acid and citric acid detectable at approximately 250 and 400 fmol, respectively. Anabasine and nornicotine had LOD_i_s of approximately 20 fmol, while the LOD_i_s of the other analyzed alkaloids were below 1 fmol. ABA, SA, JAs and CKs could be detected in the amol range, some even below 100 amol (isopentenyladenine, IP and isopentenyladenosine, IPR). The values for AXs ranged between 0.9 fmol (indole-3-acetamide, IAM) and 13 fmol (D_5_-IAA). The LOD_i_ of the GAs varied strongly, ranging from less than 1 fmol for GA_7_ up to 59 fmol for GA_29_.

Recovery rates were only determined for compounds that underwent the purification procedure (SPE and evaporation steps). For most compounds, the quantified recovery rates were above 70 % (Additional file 1: Tables S16–S22). The recovery rates of compounds decreased with the hydrophobicity of the analytes, e.g. GA_9_, GA_12_, GA_12_-aldehyde, benzylaminopurine (BAP), IP and IPR showed low recovery rates (≤15 %). Despite these low recovery rates, the high analytical sensitivity observed for IP and IPR was able to compensate for these losses and the use of isotope labeled standards ensured an accurate quantification. However, the method might be not applicable for the analysis of GA_9_, GA_12_ and BAP, except for the analysis of plant tissues that hyperaccumulate these compounds. GA_12_-aldehyde was nearly completely lost during the extraction and was therefore removed from the analysis. Similarly, we observed that the 12-oxo-phytodienoic acid (OPDA) was severely depleted from plant extracts and was also excluded from the method. These compounds might degrade during extraction or incompletely elute from the HR-XC column. Based on their high hydrophobicity they might also be removed together with other hydrophobic constituents in the first step of the sample purification (HR-X column).

For compounds that were analyzed without further purification procedure (Methods 1A, 1B, 1C and 1D) we re-analyzed samples after a prolonged storage period, to evaluate if compound stability might be a problem for their accurate determination. Between the first and the second analysis the samples stayed for a longer period of time each at 10 °C (>1 day) and −20 °C (>20 weeks), and faced additional melting-freezing cycles. Additional file 1: Table S23 shows the changes in the calculated amounts from the first analysis and their re-analysis. Only compounds are presented that were clearly detected in the samples. For most compounds (e.g., Ala, Phe, Met, Nicotine, Glucose) only minor changes were observed that might be also explained by other factors (e.g., the accuracy of peak integration). The largest changes that occurred were approximately by a factor of 2 (for shikimic acid, tryptamine and tyramine). Normally samples would not be exposed to such challenging conditions and from these results, we conclude that compound stability has only a minor influence on their accurate analysis, as long as the samples are treated appropriately, as described in the “Methods”.

High matrix effects with a more than 50 % signal reduction compared to pure standards were, except for Gly, only observed for the concentrated extracts (Methods 2B and 3) and then only for some compounds of these concentrated samples. Interestingly, many alkaloids showed an even greater sensitivity when they were measured in matrix.

Based on the slopes, the recovery rates and the matrix effects, we calculated response factors to quantify compounds with no accessible isotopically labeled standards. In case of GA_3_, the MRM settings for its double deuterated standard were determined, but for cost reasons it was excluded from the method for routine measurements.

In case of available isotopically labeled internal standards we tested only either the labeled or unlabeled compound and assumed an identical behavior for the validation parameters mentioned above. The same assumptions were made with CKs with *cis* and *trans*-isomers. The results are summarized in Additional file 1: Tables S16–S22.

Since 100 % stability of all compounds cannot be guaranteed, it is important to reduce losses by appropriate treatment of samples during sample preparation, storage and analysis as mentioned under "Methods". Additionally, storage times should be reduced as short as possible and the samples should be analyzed in a randomized order to avoid systemic errors. Errors can be greatly reduced by using isotopically labeled internal standards.

### Multiple use of SPE columns

To test if the HR-X and HR-XC columns can be re-used, we used the same set of plates three times to purify plant extracts, each with a washing and drying step between uses. Afterwards, a standardized aliquot of an herbivory-induced plant extract was purified on a set of either new or cleaned and re-used plates and measured with Method 2A, 2B or 3, respectively. We analyzed all internal standards and compared these to evaluate column-mediated effects (Additional file 1: Figure S1). During the procedure we observed that the herbivory-induced samples accumulated a green-brownish pigment that was partially retained on the HR-XC column and could not completely be removed by the washing steps. In the subsequent purification Fraction 3 also obtained a slightly darker staining. However, we observed no negative effects of this discoloration for our analysis. Even after four uses the columns achieved results comparable with those from unused columns. From these results, we conservatively recommend to use the columns up to three times, although they may retain their functionality even longer. For other tissues, these results might differ, although *N. attenuata* leaves can be assumed to represent a challenging matrix due to their intense accumulation of secondary metabolites (e.g., as shown in [22]).

### Challenges and troubleshooting

During the development of this method, a number of practical issues arose that can cause problems, misinterpretations or sample loss during extraction and analysis; these are summarized here.

Several amino acids elute very early during the chromatographic separation. To make them detectable (the signal detection of the MS roughly started 0.3 min after injection) in Methods 1A and 1B, the flow rate during the first minute after injection was lowered (slowly increasing from 250 µL/min), while a higher flow (500 µL/min) rate is used for the separation of later eluting compounds.

Some compounds share specific MRM transitions due to similar structural features and this can become a particular problem when similar compounds occur in different abundances. For example nicotine and anabasine share specific MRM transitions and elute at very similar RTs, but usually nicotine occurs in several orders of magnitude higher concentrations in *N. attenuata* leaves. Similarly, the frequently high abundant Gln can confound the analysis of Lys, and Asn results in an additional signal in the ion trace of Orn. Additionally, one constituent of the mix of isotopically labeled amino acids (most likely ^13^C_5_, ^15^N_1_-Val) interferes with the qualifier MRM transition of niacin. Cys can give a signal in the ion trace of the ^13^C_5_, ^15^N_1_-Pro quantifier. Therefore we included a specific Cys MRM transition that is not affected by ^13^C_5_, ^15^N_1_-Pro in Method 1A to ensure that the ^13^C_5_, ^15^N_1_-Pro quantification is not disturbed in a high Cys background. In some samples the ion trace for His also showed a signal from an unknown slightly earlier eluting compound.

In general, diastereomeres, such as *cis*-zeatin (*c*Z) and *trans*-zeatin (*t*Z) can be analyzed using the same ion trace. Since the included isotope labeled CK standards are in the *trans*-configuration, the use of additional qualifier traces is recommendable for the identification of the *cis*-isoform. Special care is necessary for the analysis of CK glucosides, which, depending on the type of CK, can appear as *N7*-, *N9*- and *O*-glucosides (abbreviated as ~*7*G, ~*9*G and ~*O*G, respectively). In case of zeatin glucosides the *cis* and *trans* forms additionally increase the peak number to up to 6 peaks that might appear in a single ion trace. In addition to comparing the retention times with the internal standards, the careful use of qualifiers (and especially their ratios to the quantifier) can help to correctly assign signals. Although most CK-glucosides are sufficiently separated by the UHPLC method, *c*Z*7*G and *t*Z*O*G are hardly distinguishable, despite the higher qualifier to quantifier ratio. Switching to ACN as the organic buffer (B) can influence the elution order of CK-glucosides: MeOH (presented here): *t*Z*7*G < *t*Z*O*G < *t*Z*9*G; ACN [33]: *t*Z*O*G < *t*Z*7*G < *t*Z*9*G.

Since the butenedioic acid isomers, maleic acid and fumaric acid, are inter-convertible and since their retention times overlap, we do not distinguish between these isomers. In addition, the chemical conversion between Cys and cystine should be considered.

Finally, some isotopically labelled standards share MRM transitions and could not be distinguished by their RT, i.e. Asp/Asn and Glu/Gln and were therefore designated as ^13^C_4_, ^15^N_n_-Asx and ^13^C_5_, ^15^N_n_-Glx, respectively.

Although MS/MS experiments are expected to offer a high selectivity, we still found for some quantifier traces, pronounced signals from unknown compounds that were only distinguishable by their chromatographic behavior. Examples of this type of interference were mainly found during the analysis of the concentrated extracts (Methods 2B and 3), e.g., dihydrozeatin (DHZ), DHZ riboside (DHZR), the zeatin glucosides and caffeic acid, that showed unknown signals that eluted ahead of the analyte of interest. Whether or not these signals originate from structurally similar compounds (e.g., other CKs or hydroxycinnamic acids, respectively) remains elusive, but this problem again illustrates the importance of careful signal assignments (supported e.g., by internal standards, qualifier ion traces and standard injection experiments). As an example, the MRM transition of the D_6_-IP standard shows an unknown signal (Additional file 1: Figure S2). The chromatographic separation is not sufficient to separate both signals, but since the signal remains below 5 % of the signal of the internal standard it should have only minor effects on the quantification of IP levels.

During optimization of the MS/MS parameters for the D_5_-IAA, we observed an unexpected behavior of this isotope labeled standard. Despite a single expected fragment ion with an increased m/z compared to the IAA-fragment (+)130.00 we observed three fragments with m/z of (+)133.1, (+)134.1 and (+)135.1, which probably originate from the occurrence of differentially deuterated fragments of the same precursor ion ([M + H]^+^ 181.10) (Additional file 1: Figure S3). The same effect was also observed on an API 5000 tandem mass spectrometer (data not shown) and can be seen in Figure S2B of Kojima et al. [23]. To account for this, we quantified D_5_-IAA as the sum of all three transitions. Since the background noise also sums up, we assumed a different LOD and LOQ for IAA and D_5_-IAA and determined them separately.

When using the 96-well-tubes and racks on N_2(l)_ (e.g., during aliquoting) it can happen that air components (presumably oxygen) liquefy at the bottom of the tube (Additional file 1: Figure S4). To prevent sample loss due to rapid expansion of this gas and buildup of high pressure, it is important to make small holes in the lids (e.g., with a syringe needle) and to put the samples into the freezer until the liquid evaporated completely (usually <30 min at −20 °C) before the addition of the extraction buffer.

Another drawback of the 96-well based extraction is the lower maximal centrifugation speed of the required swing-bucket rotors compared to that of fixed-angle rotors. To avoid the transfer of small particles into the UHPLC, we added an additional centrifugation step directly before analysis (after the transfer to the final 0.2 mL 96-well plates).

For sugar analysis, sorbitol is used as internal standard. It elutes between glucose and fructose and is inexpensive. However, in some plant species (e.g., apple) it naturally occurs in high amounts. Therefore, it is essential to check for the presence of sorbitol in the sample matrix and, if necessary, consider other internal standards for quantification.

### Application of the workflow

**Fig. 6:**
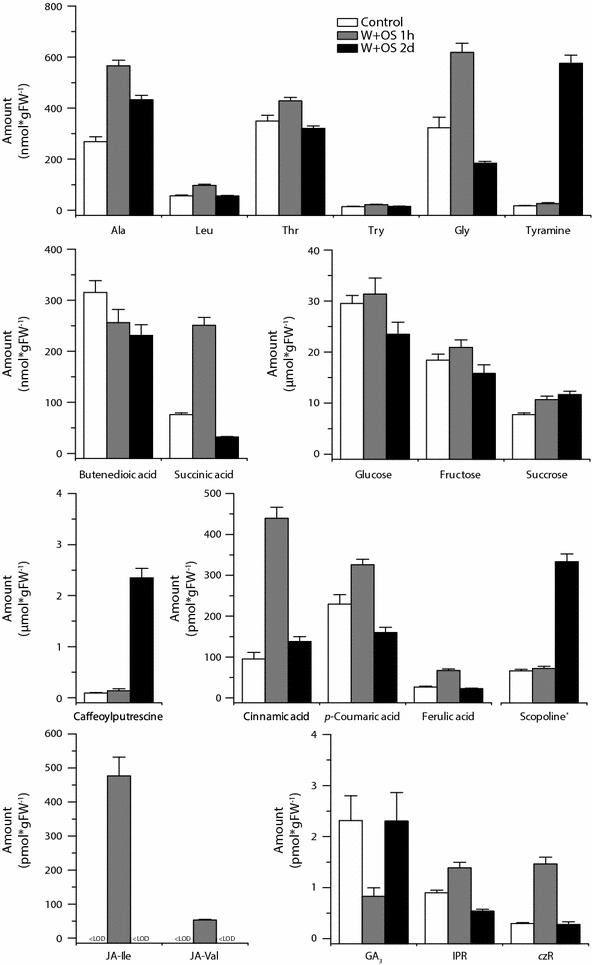
Herbivory-induced changes in metabolite levels in *N. attenuata* leaves. Changes in various plant metabolites 1 h and 2 days after wounding with a fabric pattern wheel and immediate application of *M. sexta* oral secretions to the puncture wounds (W + OS 1 h, *grey bars* and W + OS 2 days, *black bars*, respectively), as well as in untreated control leaves (Control *white bars*). The results for the other measured metabolites are shown in Additional file 1: Table S24. *Error bars* are ±SE (n = 10). *Scopoline levels are only relative quantified. *LOD* limit of detection, *FW* fresh weight

**Fig. 7:**
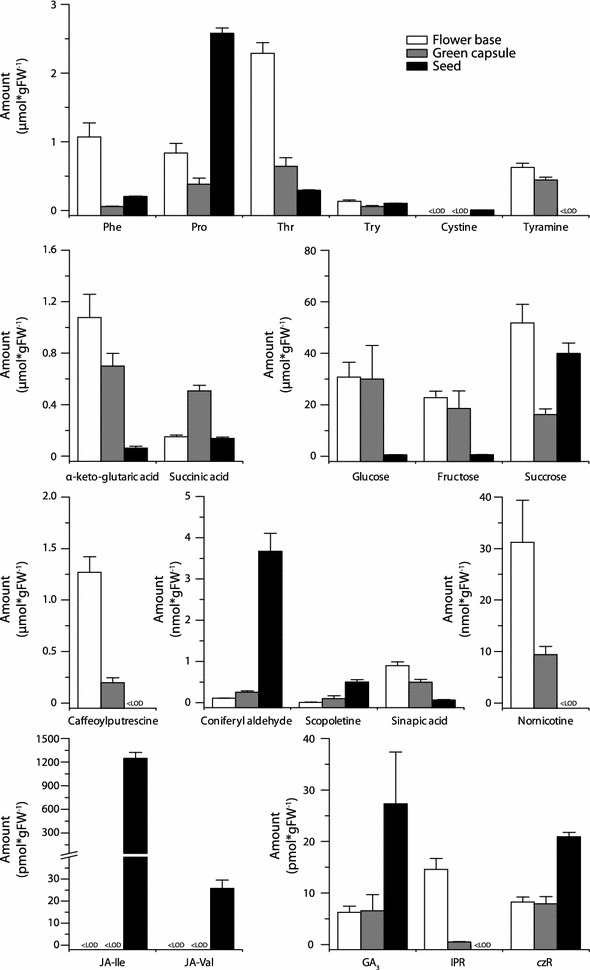
Metabolite levels during the development of *N. attenuata* seeds. Selected metabolites in the lower part of an open flower (flower base, *white bars*; flower without corolla, stamen, style and pedicel), in developing seed capsules (green capsule *grey bars*) and ripe seeds (seed *black bars*). The results for the other measured metabolites are shown in Additional file 1: Table S24. *Error bars* are ±SE (n ≥ 6). *LOD* limit of detection, *FW* fresh weight

To illustrate the applicability of the procedure, we quantified the short- and long-term metabolic changes in herbivory-induced *N. attenuata* leaves (Fig. 6) and those that occur during seed development (Fig. 7). We collected leaves 1 h and 2 days after simulated herbivory, as well as untreated controls (Fig. 6 and Additional file 1: Table S24), basal parts of individual flowers (without corolla, stamen, style and pedicel), developing green seed capsules and ripe seeds (Fig. 7 and Additional file 1: Table S24; for matrix and recovery corrected response factors used for seed and flower related tissues see Additional file 1: Table S25). Since the flower samples consisted of only approximately 10 mg tissue per sample, this sample set additionally highlights the utility of the analysis of small sample amounts.

The results nicely illustrated that both processes (herbivory response and seed development) are associated with various changes in the plant metabolism. The herbivory-induced leaves, for example, showed the characteristic induction of defense-related hormones (specifically JAs) and the accumulation of inducible defense compounds (e.g. caffeoylputrescine), but also changes of growth-related hormones and various primary metabolites (Fig. 6). Similarly, the different flower and seed-related tissues vary not only in their primary metabolites (e.g., glucose and fructose levels), but also in their abundance of defense-related compounds (caffeoylputrescine, scopoletine and nornicotine) and their phytohormone profiles (Fig. 7). The changes in various sectors of plant metabolism during both seed maturation and leaf defense highlight the value of broadly focused metabolite screens.

For a better overview, we present a visualization of the results in a hierarchical clustered heatmap (Additional file 1: Figures S5 and S6), which includes the method required for the analysis of each compound. This visualization highlights an important take-home message of this study: that compounds with similar behavior often require different analytical methods for their quantification.

Additional file 1: Figure S5 summarizes the compounds that change in abundance after simulated herbivory and their intercorrelations, from which we note the following interesting patterns that relate to previous work on the *N. attenuata* system. The *c*ZR levels were strongly correlated with the abundance of JAs, a result consistent with the work of a previous investigation [35] and underscoring that JA signaling promotes the wound-induced accumulation of *c*ZR. We note that the levels of quercetin, fructose and glucose behaved similarly throughout the samples. Quercetin was found by Roda et al. [31] to attract the mirid bug, *T. notatus*. Based on the highly variable within-plant distribution of quercetin, it was hypothesized that this flavonoid helps mirids to find suitable feeding places. The data presented here are consistent with this idea, and suggest that quercetin levels could function as a marker for the glucose and fructose content of leaves. The analysis of flower and seed-related tissues (Additional file 1: Figure S6) revealed that the levels of JA and JA-Ile were not positively correlated with the abundance of caffeoylputrescine, although it is a JA-inducible metabolite in leaf tissues [30]. This lack of correlation suggests that other co-occurring factors might modulate the JA-response. One candidate might be GA_3_, which was similarly distributed in these samples and has the potential to suppress JA-signaling [17]. In contrast, the scopoletine levels were positively correlated with JA and JA-Ile levels. JAs were shown by Sun et al. [40] to be necessary, but not sufficient, for the accumulation of scopoletine.

Such observations can be helpful to establish new hypothesis, but each of these interesting correlations require experimental manipulations to evaluate the potential causality associated with the correlation.

## Conclusions

The protocol presented here provides a convenient means of rapidly quantifying more than 100 primary and secondary metabolites, and phytohormones (AXs, JAs, GAs, CKs, ABA and SA) from small amounts (10–100 mg) of tissues. The procedure allows 192 samples to be processed in parallel (two 96 well plates) and the SPE columns were found to be useable multiple times; as such the protocol is a cost and time efficient alternative to multiple specific extraction procedures with restricted targets and untargeted metabolite screens that require time-consuming post-analysis data processing.

Due to its speed, accuracy, and broad scope of targets, we predict that the procedure will find application in the metabolic phenotyping of transgenic lines, in forward genetics screens e.g., based on genome wide association studies and recombinant inbred lines, as well as for the analysis of genetic variable plants from natural populations.

### Methods

#### Plant material and treatment

Plants from the 31st inbred generation of the ‘UT’ line of *N. attenuata* (Torr. ex S. Wats.) were germinated and grown in the glasshouse as described by Krügel et al. [24].

To simulate herbivory, we rolled a fabric pattern wheel three-times on each side of the lamina of the youngest fully-expanded leaf and immediately applied 20 µL of 1:5 water-diluted *Manduca sexta* oral secretions to the puncture wounds per leaf. Treatments were performed between 10 and 11 am. Leaves were collected after different times together with untreated controls and the leaf lamina (without midvein), were immediately shock frozen in liquid nitrogen, ground to a fine powder and stored at −80 °C until further processing. Individual plants represented the biological replicates. Flower and seed related plant tissues were collected around 12 pm from a plant in the late flowering stage. From open flowers we removed the corolla, stamen, style and pedicel and collected the remaining flower base. Additionally, green, unripe seed capsules and ripe seeds from already open seed capsules were collected. Each replicate originated from a single flower/capsule. After collection the samples were immediately flash frozen in liquid nitrogen, ground to a fine powder and stored at −80 °C until further processing.

The *M. sexta* oral secretions were collected as described by Turlings et al. [41] with the modifications of Alborn et al. [1] from *M. sexta* larvae from an in-house colony.

### Extraction and purification

For the analysis of leaf tissues, 100 mg frozen ground plant material was aliquoted into 96-well biotubes (1.1 mL individual tubes, Arctic White LLC, catalog number: AWTS-X22100) and closed with strips of 8-plug caps (Arctic White LLC, catalog number: AWSM-T100-30). For the analysis of flower and seed tissues smaller tissue amounts were used (flower base, ~10 mg; green capsule, 40–90 mg; seeds 20–30 mg). During aliquoting, the samples were kept on liquid nitrogen and subsequently stored at −20 °C until extraction (at least for 30 min). To each tube 2 steel balls (ASKUBAL, catalog number: 3 MM-G100-1.4034) were added to improve the homogenization during extraction. If applicable, samples were stored at −20 °C or kept on ice during sample preparation. For extraction, we added to each sample 800 µL precooled (−20 °C) acidified MeOH [MeOH:H_2_O:HCOOH 15:4:1 (v:v:v)] containing the internal standards (for each sample 20 ng D_4_-ABA, 20 ng D_6_-JA, 20 ng D_6_-JA-Ile, 20 ng D_6_-SA, 4.4 ng 4-MU, 3 ng D_5_-IAA, 0.25 ng D_3_-DHZ, 0.05 ng D_3_-DHZR, 0.5 ng D_5_-*t*Z, 0.1 ng D_5_-*t*ZR, 1 ng D_5_-*t*Z*7*G, 1 ng D_5_-*t*Z*9*G, 2 ng D_5_-*t*Z*O*G, 4 ng D_5_-*t*ZR*O*G, 0.1 ng D_6_-IP and 0.1 ng D_6_-IPR). The tubes were tightly sealed with a seal mate (ArctiSeal for 96 Well Tube Arrays; Arctic White LLC, catalog number: AWSM-2002RB), homogenized in a Genogrinder (60 s at 1150 strokes/min; Geno/Grinder 2000, SPEX SamplePrep) and incubated over night at −20 °C. After incubation the samples were homogenized (Genogrinder, 60 s at 1150 strokes/min), centrifuged (1913×*g* for 20 min at 4 °C) and 600 µL of the supernatant was transferred to a new vial. From the supernatant 10 µL were separately taken away as Fraction 1 (for the analysis by Methods 1A, 1B, 1C and 1D; including e.g., amino acids, alkaloids and sugars). For re-extraction of the pellet another 600 µL of extraction buffer (without internal standards) were added, the samples were mixed again (Genogrinder, 60 s at 1150 strokes/min) and incubated for another 30 min at −20 °C. After centrifugation 600 µL of the supernatant were taken and combined with the supernatant from before. The supernatants were centrifuged to remove remaining particles (1913 *g* for 20 min at 4 °C).

A 96-well HR-X column (MACHEREY-NAGEL, 96 × 25 mg, catalog number: 738530.025M) was activated with 600 µL (per well) MeOH (sucked through utilizing a Chromabond Multi 96 vacuum manifold, MACHEREY-NAGEL, catalog number: 738630.M) and preconditioned with 600 µL extraction buffer (without internal standards). Then the samples were loaded on the column and the flow-through was collected in a 96-well Nunc Deep Well Plate (Thermo Fisher Scientific, catalog number: 278752). Columns were washed each with 200 µL extraction buffer (without internal standards) and the flow-through was collected in the same Nunc plate. The samples were placed in an evaporator system and the MeOH was evaporated at 45 °C under a constant nitrogen stream (Biotage, TurboVap 96, catalog number: C103264). To ensure that most of the MeOH had evaporated, we used a calibrated pipet to evaluate that not more than approximately 350 µL liquid remained. The samples were reconstituted with 850 µL 1 N HCOOH per sample, sealed with a Nunc 96-Well Cap Mat (Thermo Fisher Scientific, catalog number: 276002) and homogenized (Genogrinder, 30 s at 1000 strokes/min).

A 96-well HR-XC column (MACHEREY-NAGEL, 96 × 25 mg, catalog number: 738540.025 M) was activated with 600 µL MeOH and preconditioned with 600 µL 1 N HCOOH. Samples were loaded to the column and the flow-through was discarded. The columns were washed with 1 mL 1 N HCOOH (discard flow-through). To elute the samples we added 1 mL acidified MeOH (0.2 N HCOOH in 80 % MeOH) and collected the flow-through in 96-well biotubes as Fraction 2 (for the analysis by Methods 2A and 2B; including e.g., JA, SA, ABA, IAA, GA). The columns were washed with 0.35 N NH_4_OH (discard flow-through). Subsequently we added 0.35 N NH_4_OH in 60 % MeOH to the columns to elute the CKs as Fraction 3 (for the analysis by Method 3).

Before analysis by Methods 1A, 1B and 1C, we diluted each 2 µL of Fraction 1 in 98 µL of a mix of ^13^C, ^15^N-labeled amino acids (Aldrich, catalog number: 487910), containing 949 fmol/µL ^13^C_3_, ^15^N_1_-Ala, ^13^C_6_, 186 fmol/µL ^15^N_4_-Arg,^13^C_4_, 1500 fmol/µL ^13^C_4_, ^15^N_n_-Asx_Asn_, 1209 fmol/µL ^13^C_4_, ^15^N_n_-Asx_Asp_, 648 fmol/µL ^13^C_5_, ^15^N_n_-Glx_Gln_, 516 fmol/µL ^13^C_5_, ^15^N_n_-Glx_Glu_, 1465 fmol/µL ^13^C_2_, ^15^N_1_-Gly, 41 fmol/µL ^13^C_6_, ^15^N_3_-His, 196 fmol/µL ^13^C_6_, ^15^N_1_-Ile, 522 fmol/µL ^13^C_6_, ^15^N_1_-Leu, 216 fmol/µL ^13^C_6_, ^15^N_2_-Lys, 8.1 fmol/µL ^13^C_5_, ^15^N_1_-Met, 255 fmol/µL ^13^C_9_, ^15^N_1_-Phe, 240 fmol/µL ^13^C_5_, ^15^N_1_-Pro, 410 fmol/µL ^13^C_3_, ^15^N_1_-Ser, 404 fmol/µL ^13^C_4_, ^15^N_1_-Thr, 191 fmol/µL ^13^C_9_, ^15^N_1_-Tyr, 210 fmol/µL ^13^C_5_, ^15^N_1_-Val (in water). For sugar analysis (Method 1D) we diluted 2 µL Fraction 1 in 998 µL of a 500 pg/µL sorbitol solution (in water). For the sugar analysis of flower base and seed samples a dilution of 1:100 and 1:50 was used instead, respectively. From Fraction 2 we separated 50 µL for the analysis by Method 2A. The rest of Fraction 2 was evaporated at 45 °C under a constant nitrogen stream until dryness and re-dissolved in 50 µL of 0.2 N HCOOH in 80 % MeOH. To insure sufficient dissolving samples were shaken (Genogrinder, 60 s at 1000 strokes/min) and sonicated (10 min in an Ultrasonic bath). Fraction 3 was also completely evaporated and re-dissolved in 50 µL 0.1 % acetic acid. To insure sufficient dissolving, samples were shaken and sonicated as mentioned before. All samples were transferred to separate 96-well PCR plates and covered with a seal mate. Samples were stored at −20 °C until analysis. Plates were centrifuged (1913×*g* for 20 min at 4 °C) before analysis.

To reduce the storage period, we recommend starting the sample analysis of Fraction 1A and 1B already during the preparation of the later fractions. Additionally, since sugars are expected to be stable under the mentioned storage conditions and since Method 1D takes the most time per sample, it should be run at latest.

During analysis samples were kept at 10 °C. We injected 1 µL sample for Methods 1A, 1C, 1D and 2A, 10 µL for Method 1B, 5 µL for Method 2B and 3 µL for Method 3. Samples should be analyzed in a randomized sequence to avoid systematic errors due to changing instrument performance or degradation (e.g., by organizing treatments/lines/… column-wise and measuring row-wise). The separation was done with a BRUKER Advance UHPLC system equipped with a Zorbax Eclipse XDB-C18 column (Methods 1A, 1B, 2A, 2B and 3), a Gemini C18 column (Method 1C) or an apHera amino (NH_2_) column (Method 1D). Solvent settings are summarized in Additional file 1: Tables S9–S15.

Analysis was performed on a Bruker Elite EvoQ Triple quad-MS equipped with a HESI (heated electrospray ionization) ion source. Source parameters are described in Additional file 1: Table S1. Samples were analyzed in MRM mode; the settings are described in Additional file 1: Tables S2–S8.

Post-run analysis was done with the ‘MS data Review’ software of the ‘Bruker MS Workstation’ (Version: 8.1.2).

### Method validation

To validate the method, we measured dilution series of analytes and isotope labeled standards. Concentrations decreased by 50 % in each dilution step and for each step we measured three independently prepared samples. Based on the results we determined the linear range, LOD, LOQ and by comparing the slopes, calculated the response factors for the respective analyte-standard combinations. We used the dilution curve method (DIN 32645) to calculate the instrument’s LOD and LOQ (LOD_i_ and LOQ_i_, respectively; on column), as well as the LOQ for the method (LOQ_m_; per sample), considering the sample volume, recovery rate, matrix effect (leaf matrix) and injection volume. If possible, for each compound the 7 lowest dilutions within the linear range and with a specific signal were used to determine LOD and LOQ. In a few cases less than 7 data points were available to define these validation parameters (indicated in Additional file 1: Tables S16–S22). For dilution curves, we used amino acids from a commercially available standard mixture. Therefore, even in cases were only the isotope labeled standard is normally included in the method, the unlabeled amino acids were used for this part of the method evaluation (for details see Additional file 1: Tables S17 and S18). The method evaluation of nicotine was performed with D_3_-nicotine to prevent disturbance with the evaluation of anabasine (D_3_-nicotine is normally not included in the method).

To determine the recovery rates during sample preparation, we used a mixture of analytes and standards and treated them as described for the sample purification procedure, including all SPE and evaporation steps, respectively. The results were compared with a mixture that was directly added to the respective buffers without additional processing steps. Due to the removal of an aliquot for the measurement by Method 2A, the compounds analyzed in Method 2B encounter a 5 % loss that was not included in the calculation of the recovery rate.

For Methods 1A, 1B, 1C and 1D we re-analyzed five exemplary samples (leaf tissue, 1 h after simulated herbivory), to determine if compound stability might influence their quantification by these methods. Before the re-analysis, the samples had been stored for more than 1 day at 10 °C, more than 20 weeks at −20 °C and experienced several melting-freezing cycles. As such, these samples experienced conditions which should challenge the stability of the analytes. To avoid disturbance by other factors such as sample evaporation, injection accuracy and instrument performance, the normalized (by internal standard, as described for a regular sample analyzes) metabolite levels were used for the comparison.

Additionally, we calculated the matrix effects on the UHPLC–MS/MS methods (see Additional file 1: Tables S16–S22) by comparing measurements from the compounds of interest with or without leaf matrix. The same leaf matrix without addition of the respective analytes was used to correct for potential background levels. The spiked matrix had at least 80 % of the strength of the pure extracts. For leaf samples the response factor was calculated based on the matrix effects observed in extracts from *N. attenuata* leaves 2 days after simulated herbivory. The samples were extracted as described under *Extraction and purification* with the modification that no internal standards were added to the extraction buffer. To reduce the errors resulting from background levels, calculations were based on the isotopically labeled standards, if available. For extracts of flower base samples, green seed capsules and seeds, particular matrix effects were determined as described above, with the exception that we used common extracts (incl. internal standards).

### SPE-column re-use

To determine if it was possible to re-use the SPE-columns, we used a HR-X and a HR-XC 96-well-SPE plate three times for the purification of plant extracts. At the end of each purification step, the plates were rinsed with 1 mL of a cleaning buffer specified for each plate (HR-X, acetone; HR-XC, 2 N NH_4_OH in 87 % acetone and subsequently with 1 mL H_2_O for pH-neutralization). Afterwards the columns were rinsed with 1 mL MeOH and dried completely before the next use. At the beginning of each purification step, the columns were first activated with 0.6 mL MeOH and conditioned with 0.6 mL of the extraction buffer or 1 N HCOOH in water as described in previous publications (e.g., [36]) for a regular first use of the column. To determine the purification capacity of the columns, we collected *N. attenuata* leaves 2 days after simulated herbivory and prepared a leaf extract in the regular way. After the re-extraction step, we combined the extracts from multiple samples and mixed them. The extract was then purified on new HR-X and HR-XC columns, as well as on the already three-times used columns. To exclude contaminations from previous uses, we did the same procedure with the re-used columns, but used pure extraction buffer without standards instead of a leaf extract.

## Additional file

10.1186/s13007-016-0130-x Quality control of multiply used solid phase extraction (SPE) columns. **Figure S2.** Overlay of the iontrace of the D_6_-IP standard and a background noise peak. **Figure S3.** Spectra and fragmentation pattern of isotope labeled and unlabeled IAA. **Figure S4.** Condensation of air components in 96-well tubes after incubation on N_2(l)_. **Figure S5.** Overview of the metabolite distribution in *N. attenuata* leaves after herbivory. **Figure S6.** Overview of the metabolite distribution in flower and seed-related tissues of *N. attenuata*. **Table S1.** Source parameters. **Table S2.** MRM-settings and retention times of analytes of Method 1A. **Table S3.** MRM-settings and retention times of analytes of Method 1B. **Table S4.** MRM-settings and retention times of analytes of Method 1C. **Table S5.** MRM-settings and retention times of analytes of Method 1D. **Table S6.** MRM-settings and retention times of analytes of Method 2A. **Table S7.** MRM-settings and retention times of analytes of Method 2B. **Table S8.** MRM-settings and retention times of analytes of Method 3. **Table S9.** Solvent settings used for Method 1A. **Table S10.** Solvent settings used for Method 1B. **Table S11.** Solvent settings used for Method 1C. **Table S12.** Solvent settings used for Method 1D. **Table S13.** Solvent settings used for Method 2A. **Table S14.** Solvent settings used for Method 2B. **Table S15.** Solvent settings used for Method 3. **Table S16.** Validation parameter for Method 1A. **Table S17.** Validation parameter for Method 1B. **Table S18.** Validation parameter for Method 1C. **Table S19.** Validation parameter for Method 1D. **Table S20.** Validation parameter for Method 2A. **Table S21.** Validation parameter for Method 2B. **Table S22.** Validation parameter for Method 3. **Table S23.** Re-analysis of selected samples after a prolonged storage time. **Table S24.** Data from two example experiments examining metabolic responses of leaves to herbivory and during seed development. **Table S25.** Response factors use for the analysis of flower and seed extracts.

## Abbreviations

4-MU: 4-methylumbelliferone
ABA: abscisic acid
AX: auxins
BAP: benzylaminopurine
CK: cytokinin
CKX: cytokinin oxidase/reductase
*c*Z: *cis*-zeatin
DHZ: dihydrozeatin
GA: gibberellin
HESI: heated electrospray ionization
HILIC: hydrophilic interaction liquid chromatography
IAA: indole-3-acetic acid
IAM: indole-3-acetamide
IP: isopentenyladenine
IPR: isopentenyladenosine
JA: jasmonic acid
JA-Ile: JA-isoleucine conjugate
LC: liquid chromatography
LOD: limit of detection
LOD_i_: instrument LOD
LOQ: limit of quantification
LOQ_i_: instrument LOQ
LOQ_m_: method LOQ
MRM: multi-reaction-monitoring
MS: mass spectrometry
OPDA: 12-oxo-phytodienoic acid
RP: reversed-phase
RT: retention times
SA: salicylic acid
SPE: solid-phase extraction
*t*Z: *trans*-zeatin
UHPLC: Ultra High Performance Liquid Chromatography
~R: riboside
~*7*G, ~*9*G and ~*O*G: *N7*-, *N9*- and *O*-glucosides
