# Supplementary material for: High-throughput quantification of more than 100 primary- and secondary-metabolites, and phytohormones by a single solid-phase extraction based sample preparation with analysis by UHPLC–HESI–MS/MS
Source: Plant Methods. 2016 May 26;12:30. doi: 10.1186/s13007-016-0130-x (PMC4882772; doi:10.1186/s13007-016-0130-x)
Supplement: Supplementary file 1 — 10.1186/s13007-016-0130-x Quality control of multiply used solid phase extraction (SPE) columns. Figure S2. Overlay of the iontrace of the D6-IP standard and a background noise peak. Figure S3. Spectra and fragmentation pattern of isotope labeled and unlabeled IAA. Figure S4. Condensation of air components in 96-well tubes after incubation on N2(l). Figure S5. Overview of the metabolite distribution in N. attenuata leaves after herbivory. Figure S6. Overview of the metabolite distribution in flower and seed-related tissues of N. attenuata. Table S1. Source parameters. Table S2. MRM-settings and retention times of analytes of Method 1A. Table S3. MRM-settings and retention times of analytes of Method 1B. Table S4. MRM-settings and retention times of analytes of Method 1C. Table S5. MRM-settings and retention times of analytes of Method 1D. Table S6. MRM-settings and retention times of analytes of Method 2A. Table S7. MRM-settings and retention times of analytes of Method 2B. Table S8. MRM-settings and retention times of analytes of Method 3. Table S9. Solvent settings used for Method 1A. Table S10. Solvent settings used for Method 1B. Table S11. Solvent settings used for Method 1C. Table S12. Solvent settings used for Method 1D. Table S13. Solvent settings used for Method 2A. Table S14. Solvent settings used for Method 2B. Table S15. Solvent settings used for Method 3. Table S16. Validation parameter for Method 1A. Table S17. Validation parameter for Method 1B. Table S18. Validation parameter for Method 1C. Table S19. Validation parameter for Method 1D. Table S20. Validation parameter for Method 2A. Table S21. Validation parameter for Method 2B. Table S22. Validation parameter for Method 3. Table S23. Re-analysis of selected samples after a prolonged storage time. Table S24. Data from two example experiments examining metabolic responses of leaves to herbivory and during seed development. Table S25. Response factors use for the analysis of flower and seed extracts. [file 13007_2016_130_MOESM1_ESM.pdf]

Additional file 1

High-throughput quantification of more than 100 primary- and secondary-metabolites, and phytohormones by a single solid-phase extraction based sample preparation with analysis by UHPLC-HESI-MS/MS

Martin Schäfer, Christoph Brütting, Ian T. Baldwin and Mario Kallenbach

Supplemental figures

Figure S1 Quality control of multiply used solid phase extraction (SPE) columns.
Figure S2 Overlay of the iontrace of the D_6-IP standard and a background noise peak.
Figure S3 Spectra and fragmentation pattern of isotope labeled and unlabeled IAA.
Figure S4 Condensation of air components in 96-well tubes after incubation on $N_{2(l)}$.
Figure S5 Overview of the metabolite distribution in *N. attenuata* leaves after herbivory.
Figure S6 Overview of the metabolite distribution in flower and seed-related tissues of *N. attenuata*.

Supplemental tables

Table S1 Source parameters
Table S2 MRM-settings and retention times of analytes of Method 1A
Table S3 MRM-settings and retention times of analytes of Method 1B
Table S4 MRM-settings and retention times of analytes of Method 1C
Table S5 MRM-settings and retention times of analytes of Method 1D
Table S6 MRM-settings and retention times of analytes of Method 2A
Table S7 MRM-settings and retention times of analytes of Method 2B
Table S8 MRM-settings and retention times of analytes of Method 3
Table S9 Solvent settings used for Method 1A
Table S10 Solvent settings used for Method 1B
Table S11 Solvent settings used for Method 1C
Table S12 Solvent settings used for Method 1D
Table S13 Solvent settings used for Method 2A
Table S14 Solvent settings used for Method 2B
Table S15 Solvent settings used for Method 3
Table S16 Validation parameter for Method 1A
Table S17 Validation parameter for Method 1B
Table S18 Validation parameter for Method 1C
Table S19 Validation parameter for Method 1D
Table S20 Validation parameter for Method 2A
Table S21 Validation parameter for Method 2B
Table S22 Validation parameter for Method 3
Table S23 Re-analysis of selected samples after a prolonged storage time
Table S24 Data from two example experiments examining metabolic responses of leaves to herbivory and during seed development
Table S25 Response factors use for the analysis of flower and seed extracts.

Supplemental figures

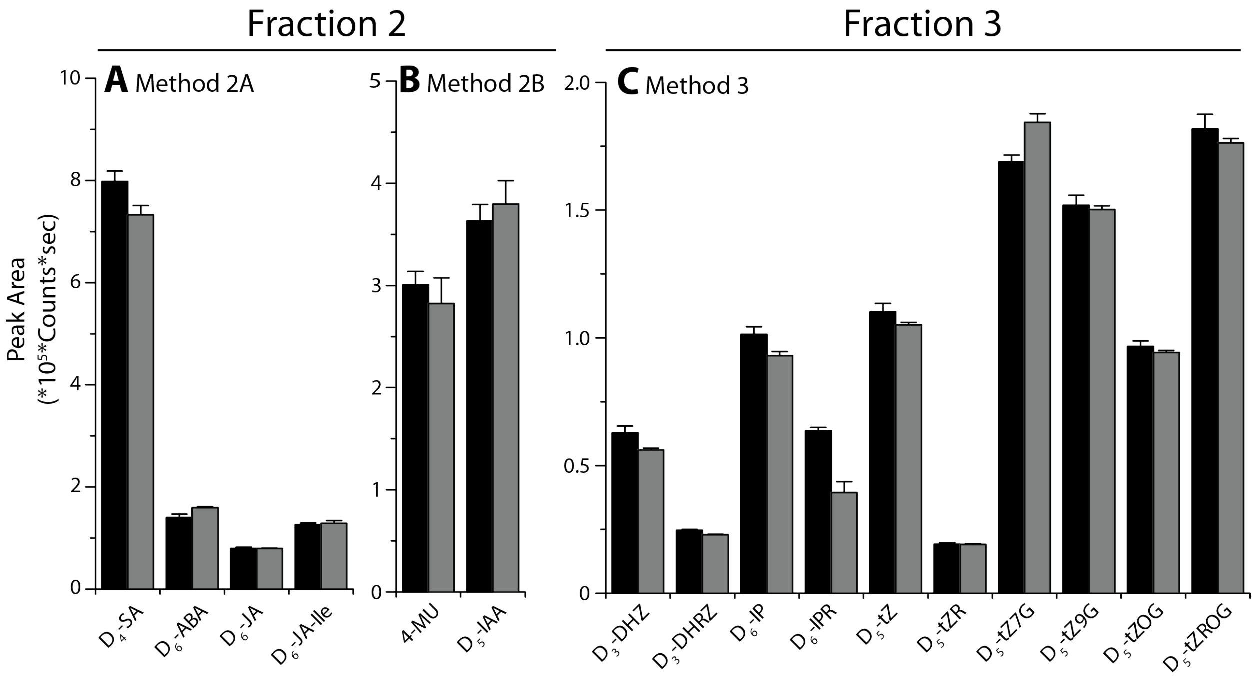

Figure S1 Quality control of multiply used solid phase extraction (SPE) columns.

Average signal intensity (±SE; n=4) of isotopically labeled phytohormone standards and 4-methylumbelliferone (4-MU) extracted and purified together with leaf samples two days after simulated herbivory. The purification was done either on a new set of SPE columns (grey bars) or on columns that were used 3 times previously (solid bars). A washing step was included between each use.

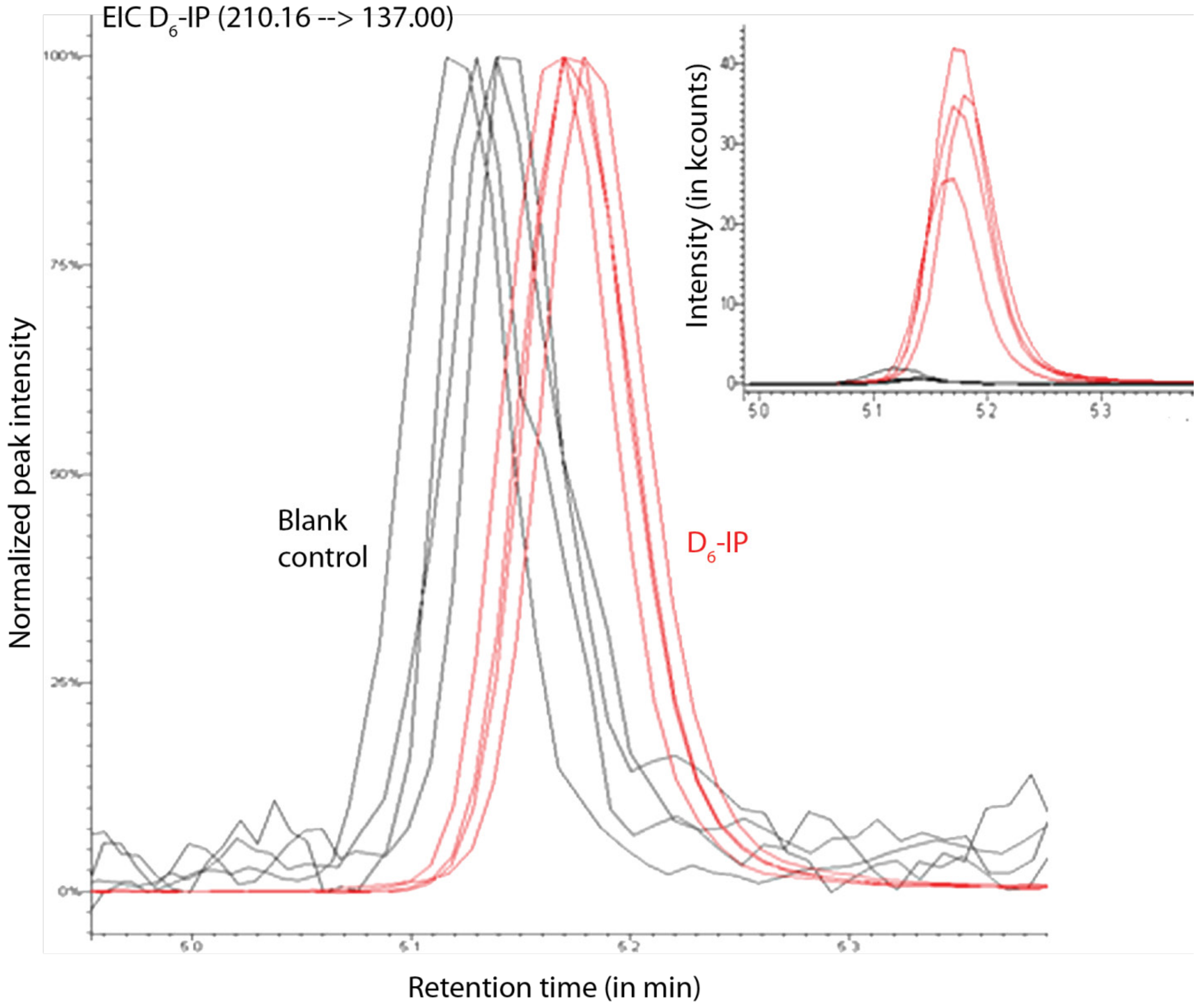

Figure S2 Overlay of the iontrace of the D_6-IP standard and a background noise peak.

D_6-IP trace (+)210.16 →137.00 from an plant tissue extract and from an equally treated negative control (no tissue and no internal standards added). The figure shows an overlay from each four independent negative controls (black lines) and plant extracts (red lines). In the main chart all samples were normalized to the most intense signal for (+)210.16 →137.00 within the RT time window (depicted as 100%). The inset shows the absolute signal intensity (kilo counts per second, kCps).

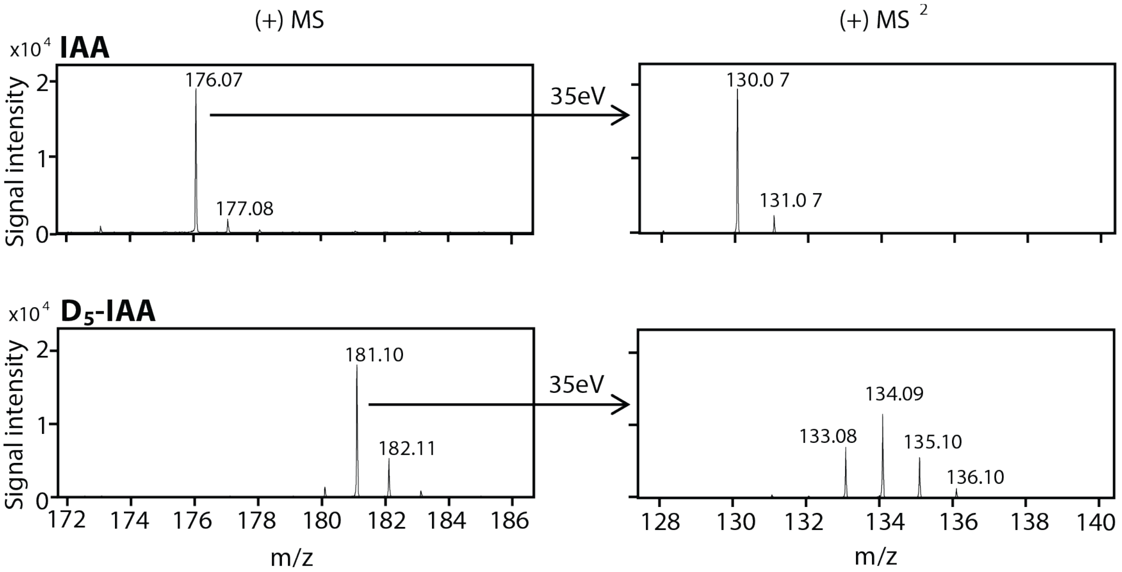

Figure S3 Spectra and fragmentation pattern of isotope labeled and unlabeled IAA.

The MS and MS/MS spectra for IAA and D_5-IAA were acquired on a micrOTOF-Q (Bruker) equipped with an ESI-source. The measurements were performed in the positive ionization mode. For MS/MS analysis the ions 176.07 and 181.10 were fragmented with 35eV.

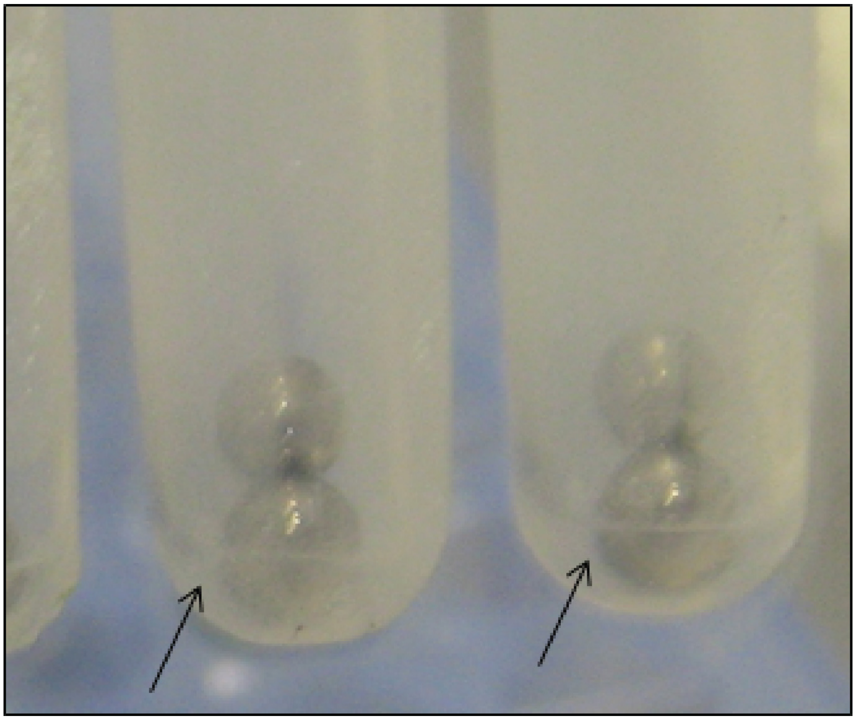

Figure S4 Condensation of air components in 96-well tubes after incubation on $N_{2(l)}$.

Black arrows indicate the liquid (presumably oxygen) that accumulates at the bottom of the tubes after short incubation on $N_{2(l)}$. The time until visible liquid accumulation varies depending on how deep the tubes are covered with $N_{2(l)}$. The tubes were closed with 8-plug caps.

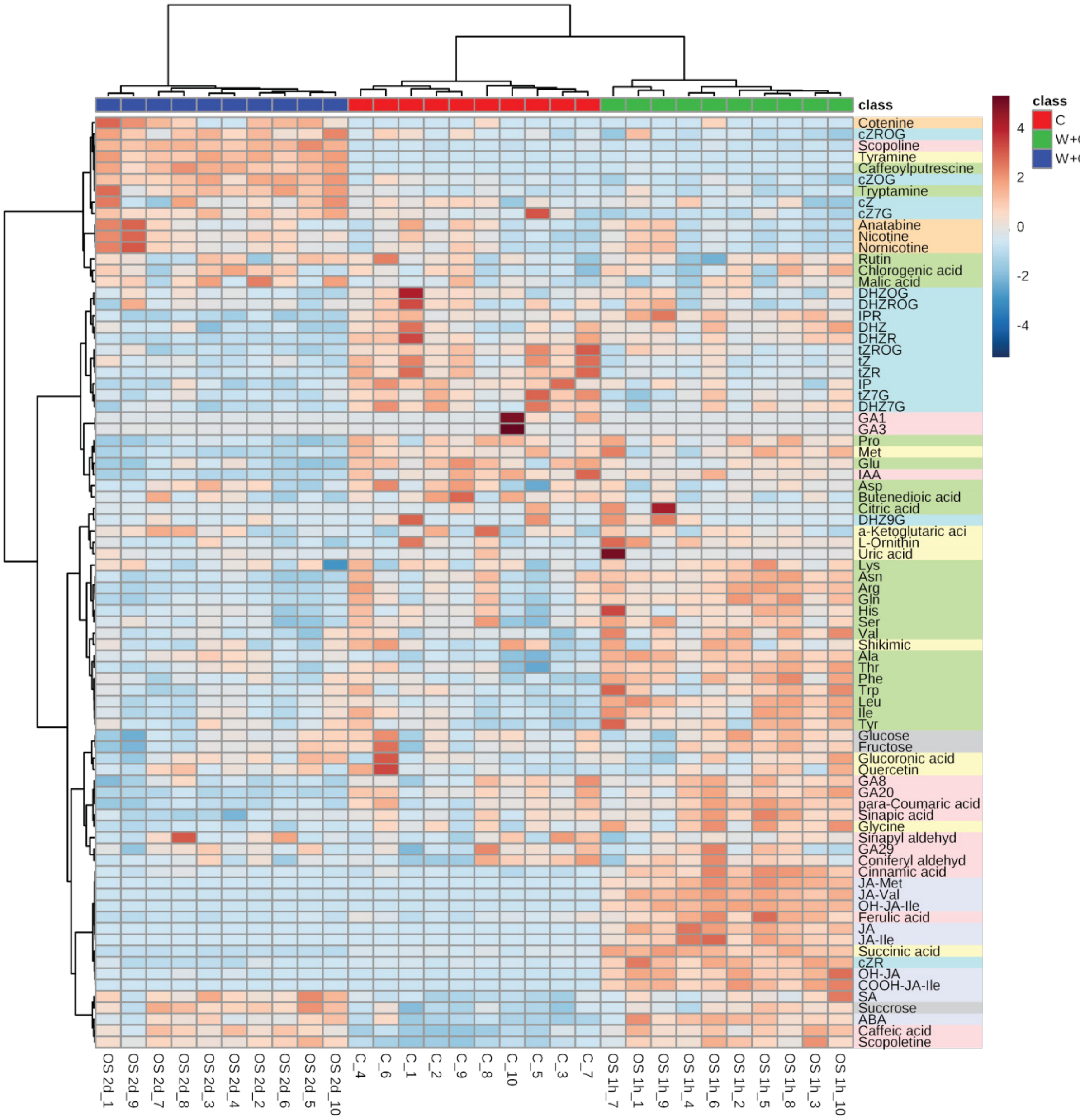

Figure S5 Overview of the metabolite distribution in *N. attenuata* leaves after herbivory.

Heatmap with hierarchical clustering based on Pearson distance measure and Ward clustering algorithm (without data filtering or additional data normalization). The Figure was generated with MetaboAnalyst 3.0 (http://www.metaboanalyst.ca, access date: 13.04.2016). Metabolites were analyzed in leaf tissues 1 h and 2 d after artificial herbivory (W+OS 1h and W+OS 2d, respectively), as well as in untreated control samples (C). Individual biological replicates are presented in rows and compounds in

columns. The absolute values of the metabolites are shown in Table S24. Only metabolites that appeared in at least one of the treatment types are shown. The *background color* of compound names indicates the specific MS method they are part of (Method 1A *green*, 1B *yellow*, 1C *orange*, 1D *grey*, 2A *blue*, 2B *pink*, 3 *light blue*).

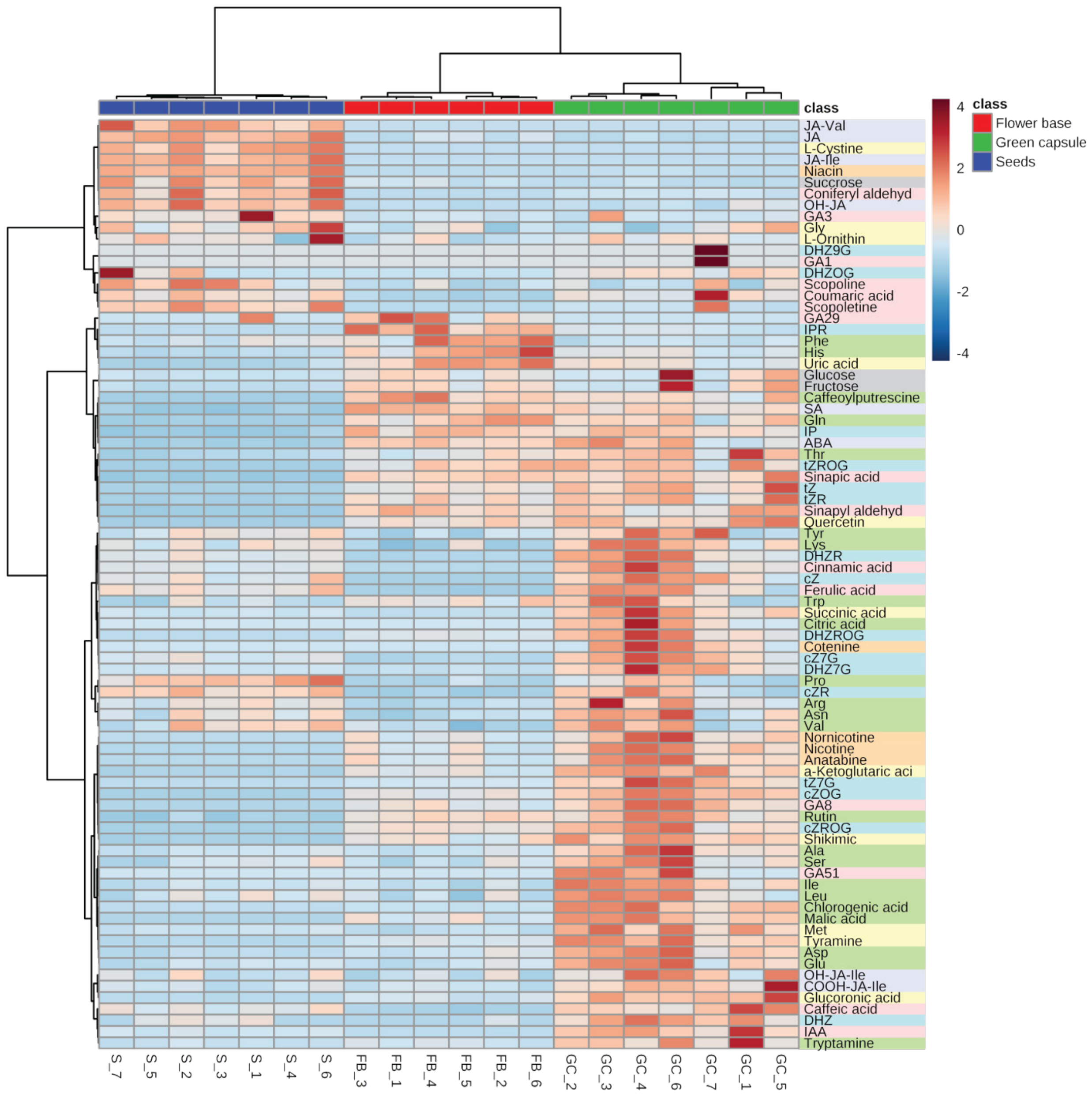

Figure S6 Overview of the metabolite distribution in flower and seed-related tissues of *N. attenuata*.

Heatmap with hierarchical clustering based on Pearson distance measure and Ward clustering algorithm (without data filtering or additional data normalization). The Figure was generated with MetaboAnalyst 3.0 (http://www.metaboanalyst.ca, access date: 13.04.2016). Metabolites were analyzed in the lower part of an open flower (Flower base; flower without corolla, stamen, style and pedicel), in developing seed capsules

(Green capsule) and ripe seeds (Seeds). Individual biological replicates are presented in rows and compounds in columns. The absolute values of the metabolites are shown in Table S24. Only metabolites that appeared in at least one of the tissue types are shown. The *background color* of compound names indicates the specific MS method they are part of (Method 1A *green*, 1B *yellow*, 1C *orange*, 1D *grey*, 2A *blue*, 2B *pink*, 3 *light blue*).

Supplemental tables

Table S1 Source parameters

Method	$Spray_{neg}$ [V]	$Spray_{pos}$ [V]	Cone gas[a]	Probe gas[a]	Nebulizer gas[b]
1A	4000	4500	35/350°C	60/500°C	60
1B	4000	4500	35/350°C	60/500°C	60
1C	0	4500	25/350°C	35/300°C	35
1D	4000	0	35/350°C	60/500°C	60
2A	4500	4500	35/350°C	60/475°C	60
2B	4000	4500	35/350°C	60/475°C	60
3	0	4000	35/350°C	60/500°C	60

[a] Flow [arbitrary units]/temperature

[b] Flow [arbitrary units]

Table S2 MRM-settings and retention times of analytes of Method 1A

Analyte	RT [min]	Q1 [m/z] →	Q3 [m/z][a,b]	CE [V][a]	Standard[c]
Ala	0.62	(+)90.06 →	44.40	-9	$^{13}C_3$, $^{15}N_1$-Ala
Arg	0.63	(+)175.12 →	70.30	-19	$^{13}C_6$, $^{15}N_4$-Arg
Asn	0.63	(+)133.06 →	74.20	-12	$^{13}C_4$, $^{15}N_n$-Asx_{Asn}[d]
Asp	0.62	(+)134.04 →	74.20	-12	$^{13}C_4$, $^{15}N_n$-Asx_{Asp}[d]
Caffeoylputresine	2.16 & 2.27	(+)251.14 →	163.00	-16	$^{13}C_9$, $^{15}N_1$-Tyr (0.30)
		(+)251.14 →	89.20	-11	
Chlorogenic acid	2.45	(+)355.10 →	163.00	-11	$^{13}C_9$, $^{15}N_1$-Tyr (0.92)
		(+)355.10 →	145.00	-26	
Citric acid	1.11	(-)191.02 →	111.10	8	$^{13}C_9$, $^{15}N_1$-Tyr (47)
		(-)191.02 →	87.10	13	
Butenedioic acid (fumaric acid & maleic acid)	1.05	(-)115.00 →	71.20	4	$^{13}C_9$, $^{15}N_1$-Tyr (1.6)
Gln	0.62	(+)147.08 →	130.10	-6	$^{13}C_5$, $^{15}N_n$-Glx_{Gln}[d]
Glu	0.64	(+)148.06 →	84.20	-14	$^{13}C_5$, $^{15}N_n$-Glx_{Glu}[d]
His	0.62	(+)156.08 →	110.10	-11	$^{13}C_6$, $^{15}N_3$-His
Ile	1.37	(+)132.10 →	86.20	-8	$^{13}C_6$, $^{15}N_1$-Ile
		(+)132.10 →	69.3	-14	
Leu	1.46	(+)132.10 →	86.20	-8	$^{13}C_6$, $^{15}N_1$-Leu
Lys	0.60	(+)147.11 →	84.20	-13	$^{13}C_6$, $^{15}N_2$-Lys
Malic acid	0.80	(-)133.01 →	115.10	5	$^{13}C_9$, $^{15}N_1$-Tyr[e]
		(-)133.01 →	71.20	11	
Phe	2.18	(+)166.09 →	120.10	-10	$^{13}C_9$, $^{15}N_1$-Phe
Pro	0.69	(+)116.07 →	70.20	-12	$^{13}C_5$, $^{15}N_1$-Pro
Rutin	2.80	(+)611.16 →	302.90	-23	$^{13}C_9$, $^{15}N_1$-Tyr (2.6)
		(+)611.16 →	465.00	-10	
Ser	0.61	(+)106.05 →	60.30	-8	$^{13}C_3$, $^{15}N_1$-Ser
Thr	0.63	(+)120.07 →	74.20	-8	$^{13}C_4$, $^{15}N_1$-Thr
Trp	2.37	(+)205.10 →	188.00	-7	$^{13}C_9$, $^{15}N_1$-Phe (3.3)
		(+)205.10 →	146.00	-15	

Tyr	1.35	(+)182.08 → 136.10	-11	$^{13}C_9$, $^{15}N_1$-Tyr
Tyramine	1.58	(+)138.10 → 121.10	-7	$^{13}C_9$, $^{15}N_1$-Tyr (0.93)
		(+)138.10 → 77.20	-25	
Val	0.83	(+)118.09 → 72.30	-8	$^{13}C_5$, $^{15}N_1$-Val
$^{13}C_3$, $^{15}N_1$-Ala	0.60	(+)94.06 → 47.40	-9	
$^{13}C_6$, $^{15}N_4$-Arg	0.62	(+)185.12 → 75.30	-19	
$^{13}C_4$, $^{15}N_n$-Asx	0.61	(+)139.05 → 77.20	-12	
$^{13}C_5$, $^{15}N_n$-Glx	0.62	(+)154.07 → 89.20	-14	
$^{13}C_6$, $^{15}N_3$-His	0.60	(+)165.09 → 118.10	-11	
$^{13}C_6$, $^{15}N_1$-Ile	1.32	(+)139.12 → 92.20	-8	
$^{13}C_6$, $^{15}N_1$-Leu	1.42	(+)139.12 → 92.20	-8	
$^{13}C_6$, $^{15}N_2$-Lys	0.60	(+)155.12 → 90.20	-13	
$^{13}C_9$, $^{15}N_1$-Phe	2.14	(+)176.11 → 129.10	-10	
$^{13}C_5$, $^{15}N_1$-Pro[f]	0.67	(+)122.08 → 75.30	-12	
		(+)122.03 → 59.20	-19	
$^{13}C_3$, $^{15}N_1$-Ser	0.60	(+)110.06 → 63.30	-8	
$^{13}C_4$, $^{15}N_1$-Thr	0.62	(+)125.07 → 78.20	-8	
$^{13}C_9$, $^{15}N_1$-Tyr	1.34	(+)192.10 → 145.10	-11	
$^{13}C_5$, $^{15}N_1$-Val	0.81	(+)124.10 → 77.30	-8	

RT: retention time

CE: collision energy

[a] Qualifiers are depicted in grey

[b] Resolution: Q1: 0.7, Q3: 2

[c] Incl. matrix (leaf) and recovery corrected response factor in brackets

[d] Asx and Glx handled as Asn, Asp, Glu or Gln equivalents, respectively

[e] Relative quantification

[f] grey depicted transition is specific for Cys, which could interfere with the $^{13}C_5$, $^{15}N_1$-Pro analysis

Table S3 MRM-settings and retention times of analytes of Method 1B

Analyte	RT [min]	Q1 [m/z] →	Q3 [m/z][a,b]	CE [V][a]	Standard[c]
α-Ketoglutaric acid	0.88	(-)145.01 →	57.30	8	$^{13}C_9$, $^{15}N_1$-Tyr (19)
		(-)145.01 →	101.10	4	
Cystine	0.61	(+)241.03	152.00	-10	$^{13}C_9$, $^{15}N_1$-Tyr (5.5)
		(+)241.03 →	74.20	-21	
Glucuronic acid	0.63	(-)193.04 →	73.20	10	$^{13}C_9$, $^{15}N_1$-Tyr (34)
		(-)193.04 →	113.10	6	
Gly	0.61	(+)76.04 →	30.50	-8	$^{13}C_2$, $^{15}N_1$-Gly
Met	0.94	(+)150.06 →	56.30	-14	$^{13}C_5$, $^{15}N_1$-Met
Ornithine	0.58	(+)133.10 →	70.30	-14	$^{13}C_6$, $^{15}N_2$-Tyr (2.4)
		(+)133.10 →	116.10	-6	
Quercetin	3.05	(-)301.04 →	151.00	18	$^{13}C_9$, $^{15}N_1$-Tyr (5.8)
		(-)301.04 →	179.00	15	
Shikimic acid	0.82	(-)173.05 →	93.10	8	$^{13}C_9$, $^{15}N_1$-Tyr (112)
		(-)173.05 →	111.10	4	
Succinic acid	1.41	(-)117.02 →	73.20	7	$^{13}C_9$, $^{15}N_1$-Tyr (1.4)
		(-)117.02 →	99.10	5	
Tryptamine	2.37	(+)161.11 →	144.10	-8	$^{13}C_9$, $^{15}N_1$-Tyr (0.13)
		(+)161.11 →	117.10	-24	
Uracil	0.93	(-)111.02 →	42.40	10	$^{13}C_9$, $^{15}N_1$-Tyr (6.5)
		(-)111.02 →	80.10	17	
Uric acid	1.10	(-)167.02 →	124.10	10	$^{13}C_9$, $^{15}N_1$-Tyr (86)
		(-)167.02 →	42.40	16	
$^{13}C_2$, $^{15}N_1$-Gly	0.60	(+)79.04 →	32.50	-8	
$^{13}C_5$, $^{15}N_1$-Met	0.93	(+)156.07 →	60.30	-14	
$^{13}C_9$, $^{15}N_1$-Tyr	1.34	(+)192.10 →	145.10	-11	
Tyr[d]	1.35	(+)182.08 →	136.10	-11	

RT: retention time

CE: collision energy

[a] Qualifiers are depicted in grey

[b] Resolution: Q1: 0.7, Q3: 2

[c] Incl. matrix and recovery corrected response factor for leaf material in brackets

[d] Usually not included in the method (just for method evaluation of $^{13}C_9$, $^{15}N_1$-Tyr)

Table S4 MRM-settings and retention times of analytes of Method 1C

Analyte	RT [min]	Q1 [m/z] → Q3 [m/z][a,b]	CE [V][a]	Standard[c]
Anabasine	4.17	(+)163.12 → 118.10	-20	$^{13}C_9$, $^{15}N_1$-Phe (3.9)
		(+)163.12 → 92.00	-20	
Anatabine	3.74	(+)161.11 → 144.10	-11	$^{13}C_9$, $^{15}N_1$-Phe (0.13)
		(+)161.11 → 80.20	-22	
		(+)161.11 → 107.10	-10	
Cotinine	2.75	(+)177.10 → 80.20	-21	$^{13}C_9$, $^{15}N_1$-Phe (0.13)
		(+)177.10 → 98.00	-20	
Niacin	0.37	(+)124.04 → 80.20	-19	$^{13}C_9$, $^{15}N_1$-Phe (5.8)
		(+)124.04 → 78.00	-18	
Nicotine	4.27	(+)163.12 → 130.10	-17	$^{13}C_9$, $^{15}N_1$-Phe (0.38)
Nornicotine	3.69	(+)149.11 → 80.20	-18	$^{13}C_9$, $^{15}N_1$-Phe (0.083)
		(+)149.11 → 130.10	-14	
		(+)149.11 → 117.10	-22	
Scopolamine	4.37	(+)304.15 → 138.10	-19	$^{13}C_9$, $^{15}N_1$-Phe (0.063)
		(+)304.15 → 156.10	-14	
		(+)304.15 → 103.10	-33	
$^{13}C_9$, $^{15}N_1$-Phe	0.62	(+)176.11 → 129.10	-10	
Phe[d]	0.62	(+)166.09 → 120.10	-10	

RT: retention time

CE: collision energy

[a] Qualifiers are depicted in grey

[b] Resolution: Q1: 0.7, Q3: 2

[c] Incl. matrix (leaf) and recovery corrected response factor in brackets

[d] Usually not included in the method (just for method evaluation of $^{13}C_9$, $^{15}N_1$-Phe)

Table S5 MRM-settings and retention times of analytes of Method 1D

Analyte	RT [min]	Q1 [m/z] →	Q3 [m/z][a,b]	CE [V][a]	Standard[c]
Cellubiose	7.44	(-)341.11 →	161.10	3	Sorbitol (5.8)
		(-)341.11 →	101.10	11	
Fructose	4.72	(-)179.06 →	89.10	5	Sorbitol (0.69)
		(-)179.06 →	71.20	10	
Glucose	5.71	(-)179.06 →	89.10	5	Sorbitol (0.95)
		(-)179.06 →	71.20	7	
Rhamnose	3.72	(-)163.06 →	59.30	9	Sorbitol (1.4)
		(-)163.06 →	103.10	4	
Sucrose	6.92	(-)341.11 →	89.20	17	Sorbitol (2.8)
		(-)341.11 →	179.00	10	
Sorbitol	5.07	(-)181.07 →	59.30	16	
		(-)181.07 →	71.20	14	

RT: retention time

CE: collision energy

[a] Qualifiers are depicted in grey

[b] Resolution: Q1: 0.7, Q3: 2

[c] Incl. matrix (leaf) and recovery corrected response factor in brackets

Table S6 MRM-settings and retention times of analytes of Method 2A

Analyte	RT [min]	Q1 [m/z] →	Q3 [m/z][a,b]	CE [V][a]	Standard[c]
ABA	2.26	(-)263.13 →	153.00	9	D_4-ABA
COOH-JA-Ile	2.22	(-)352.00 →	130.00	19	D_6-JA-Ile[d]
JA	2.52	(-)209.12 →	59.00	12	D_6-JA
JA-Ile	2.81	(-)322.20 →	130.00	19	D_6-JA-Ile
JA-Met	2.60	(-)340.16 →	148.10	13	D_6-JA-Ile (1.83)
		(-)340.16 →	47.30	24	
JA-Phe	2.79	(-)356.19 →	164.10	16	D_6-JA-Ile (1.54)
		(-)356.19 →	147.10	26	
JA-Trp	2.70	(-)395.20 →	203.11	15	D_6-JA-Ile[d]
		(-)395.20 →	245.05	10	
JA-Val	2.64	(-)308.19 →	116.10	16	D_6-JA-Ile (0.49)
		(-)308.19 →	158.10	10	
OH-JA	1.91	(-)225.11 →	59.00	12	D_6-JA[d]
OH-JA-Ile	2.25	(-)338.20 →	130.00	19	D_6-JA-Ile[d]
SA	2.25	(-)137.02 →	93.00	15	D_6-SA
D_4-ABA	2.26	(-)269.17 →	159.00	10	
D_6-JA	2.51	(-)215.15 →	59.00	10	
D_6-JA-Ile	2.71 & 2.80	(-)328.24 →	130.00	19	
D_6-SA	2.24	(-)141.05 →	97.00	15	

RT: retention time

CE: collision energy

[a] Qualifiers are depicted in grey

[b] Resolution: Q1: 0.7, Q3: 2

[c] Incl. matrix (leaf) and recovery corrected response factor in brackets

[d] Relative quantification

Table S7 MRM-settings and retention times of analytes of Method 2B

Analyte	RT [min]	Q1 [m/z] →	Q3 [m/z][a,b]	CE [V][a]	Standard[c]
Caffeic acid	1.77	(-)179.04 →	135.10	11	4-MU (0.47)
		(-)179.04 →	107.10	18	
Cinnamic acid	4.43	(-)147.05 →	103.10	7	4-MU (4.3)
		(-)147.05 →	77.20	19	
Coniferyl aldehyde	2.48	(+)179.07 →	147.00	-11	4-MU (0.41)
		(+)179.07 →	91.10	-24	
para-Coumaric acid	2.17 & 2.28	(-)163.04 →	119.10	10	4-MU (0.12)
		(-)163.04 →	93.20	26	
meta-Coumaric acid	2.56	(-)163.04 →	119.10	10	4-MU (0.14)
		(-)163.04 →	93.20	26	
ortho-Coumaric acid	3.11	(-)163.04 →	119.10	10	4-MU (0.13)
		(-)163.04 →	93.20	26	
Ferulic acid	2.26 & 2.48	(-)193.05 →	134.10	11	4-MU (0.63)
		(-)193.05 →	178.00	8	
Fraxetin	1.87	(-)207.03 →	191.90	11	4-MU (0.39)
		(-)207.03 →	108.10	19	
GA_1	2.39	(-)347.15 →	259.00	17	4-MU (1.59)
		(-)347.15 →	303.00	17	
GA_3	2.24	(-)345.14 →	143.00	26	4-MU (1.0)
		(-)345.14 →	239.00	13	
GA_4	6.84	(-)331.16 →	243.00	13	4-MU (0.58)
		(-)331.16 →	257.00	22	
GA_5	4.38	(-)329.14 →	145.00	19	4-MU (2.31)
		(-)329.14 →	241.10	8	
		(-)329.14 →	285.00	10	
GA_7	6.42	(-)329.14 →	223.10	14	4-MU (0.083)
GA_8	1.68	(-)363.15 →	275.00	13	4-MU (16)
GA_9	8.24	(-)315.16 →	271.00	19	4-MU (35)

GA_{12}	9.74	(-)331.19 → 313.00	26	4-MU (88)
		(-)331.19 → 269.00	32	
GA_{12ald}	9.91	(-)315.20 → 271.10	21	
		(-)315.20 → 163.00	26	
GA_{13}	4.86	(-)377.16 → 303.00	20	4-MU (0.68)
		(-)377.16 → 359.00	11	
		(-)377.16 → 215.10	31	
GA_{20}	4.63	(-)331.16 → 287.00	19	4-MU (5.6)
		(-)331.16 → 225.00	20	
GA_{24}	7.99	(-)345.17 → 257.00	24	4-MU (10)
		(-)345.17 → 327.00	19	
GA_{29}	1.77	(-)347.15 → 259.10	10	4-MU (71)
		(-)347.15 → 303.00	13	
		(-)347.15 → 163.00	19	
GA_{44}	5.18	(-)345.17 → 301.00	18	4-MU (9.0)
		(-)345.17 → 273.00	21	
		(-)345.17 → 255.00	22	
GA_{51}	5.67	(-)331.16 → 243.00	9	4-MU (0.65)
		(-)331.16 → 287.00	15	
IAA	2.79	(+)176.07 → 130.00	-14	D_5-IAA
IA-Ala	2.37	(+)247.11 → 130.10	-15	D_5-IAA (0.46)
		(+)247.11 → 90.20	-10	
IAM	1.89	(+)175.09 → 130.10	-13	D_5-IAA (0.51)
		(+)175.09 → 77.20	-40	
IBA	4.64	(+)204.10 → 186.00	-9	D_5-IAA (1.5)
		(+)204.10 → 130.10	-23	
Scopoletine	2.13	(+)193.05 → 178.00	-21	4-MU (1.2)
		(+)193.05 → 133.00	-19	
Scopolin	1.58	(+)355.10 → 193.00	-10	4-MU[d]
Sinapic acid	2.25 & 2.49	(+)225.08 → 207.00	-7	4-MU (0.47)
		(+)225.08 → 175.00	-13	

Sinapyl aldehyde	2.44	(-)207.07 → 192.00	10	4-MU[d]
		(-)207.07 → 177.00	17	
4-MU	2.9	(+)177.05 → 77.20	-32	
		(+)177.05 → 121.00	-21	
D_5-IAA [e]	2.75	(+)181.10 → 135.00	-14	
		(+)181.10 → 134.00	-14	
		(+)181.10 → 133.00	-14	
D_2-GA_3 [f]	2.24	(-)347.14 → 143.00	26	
		(-)347.14 → 241.00	13	

RT: retention time

CE: collision energy

4-MU: 4-methylumbelliferone

[a] Qualifiers are depicted in grey

[b] Resolution: Q1: 0.7, Q3: 2

[c] Incl. matrix (leaf) and recovery corrected response factor in brackets

[d] Relative quantification

[e] Analyzed as the sum of all three transitions

[f] Usually not included in the method (just for method evaluation of GA_3)

Table S8 MRM-settings and retention times of analytes of Method 3

Analyte	RT [min]	Q1 [m/z] → Q3 [m/z][a,b]	CE [V][a]	Standard[c]
BAP	5.36	(+)226.11 → 91.10	-20	D_6-IP (2.0)
		(+)226.11 → 65.20	-44	
*c*Z	2.53	(+)220.12 → 136.60	-16	D_5-*t*Z
		(+)220.12 → 148.30	-16	
*c*ZR	4.45	(+)352.16 → 220.30	-16	D_5-*t*ZR
		(+)352.16 → 136.00	-25	
*c*Z*7*G	2.16	(+)382.17 → 220.20	-19	D_5-*t*Z*7*G
		(+)382.17 → 136.00	-30	
*c*Z*9*G	2.66	(+)382.17 → 220.20	-19	D_5-*t*Z*9*G
		(+)382.17 → 136.00	-30	
*c*Z*O*G	2.34	(+)382.17 → 220.20	-19	D_5-*t*Z*O*G
		(+)382.17 → 136.00	-30	
*c*ZR*O*G	4.02	(+)514.21 → 382.10	-17	D_5-*t*ZR*O*G
DHZ	2.36	(+)222.14 → 136.30	-16	D_3-DHZ
DHZR	4.16	(+)354.18 → 222.10	-16	D_3-DHZR
DHZ*7*G	2.23 & 2.30	(+)384.19 → 222.00	-26	D_5-*t*Z*9*G (6.2)
DHZ*9*G	2.46	(+)384.19 → 222.00	-26	D_5-*t*Z*9*G (2.3)
DHZ*O*G	2.40	(+)384.19 → 222.00	-26	D_5-*t*Z*9*G(5.4)
DHZR*O*G	3.98	(+)516.21 → 222.20	-28	D_3-*t*ZR*O*G (1.1)
		(+)516.21 → 384.00	-20	
IP	5.18	(+)204.12 → 136.00	-14	D_6-IP
IPR	6.10	(+)336.17 → 204.30	-12	D_6-IPR
IP*9*G	5.17	(+)366.18 → 204.00	-16	D_6-IP (0.21)
		(+)366.18 → 136.00	-28	
		(+)366.18 → 148.00	-24	
*t*Z	2.25	(+)220.12 → 136.60	-16	D_5-*t*Z
		(+)220.12 → 148.30	-16	
*t*ZR	4.04	(+)352.16 → 220.30	-16	D_5-*t*ZR
		(+)352.16 → 136.00	-25	

*t*Z*7*G	2.04	(+)382.17 → 220.20	-19	D_5-*t*Z*7*G
		(+)382.17 → 136.00	-30	
*t*Z*9*G	2.41	(+)382.17 → 220.20	-19	D_5-*t*Z*9*G
		(+)382.17 → 136.00	-30	
*t*Z*O*G	2.13	(+)382.17 → 220.20	-19	D_5-*t*Z*O*G
		(+)382.17 → 136.00	-30	
*t*ZR*O*G	3.62	(+)514.21 → 382.10	-17	D_5-*t*ZR*O*G
D_3-DHZ	2.33	(+)225.15 → 136.60	-16	
D_3-DHZR	4.10	(+)357.19 → 225.30	-16	
D_6-IP	5.11	(+)210.16 → 137.00	-14	
D_6-IPR	6.07	(+)342.20 → 210.00	-12	
D_5-*t*Z	2.22	(+)225.15 → 136.60	-16	
D_5-*t*ZR	3.98	(+)357.19 → 225.30	-16	
D_5-*t*Z*7*G	2.02	(+)387.20 → 225.00	-19	
D_5-*t*Z*9*G	2.38	(+)387.20 → 225.00	-19	
D_5-*t*Z*O*G	2.11	(+)387.20 → 225.00	-19	
D_5-*t*ZR*O*G	3.58	(+)519.24 → 387.10	-17	

RT: retention time

CE: collision energy

[a] Qualifiers are depicted in grey

[b] Resolution: Q1: 0.7, Q3: 2

[c] Incl. matrix (leaf) and recovery corrected response factor in brackets

Table S9 Solvent settings used for Method 1A

Time [min]	A[a] [%]	B[b] [%]	Flow [µL/min]
0.00	99	1	250
1.00	99	1	500
2.70	0	100	500
3.70	0	100	500
3.90	99	1	250
4.90	99	1	250

[a] 0.1% ($^{v}/_{v}$) ACN, 0.05% ($^{v}/_{v}$) HCOOH in water

[b] MeOH

Table S10 Solvent settings used for Method 1B

Time [min]	A[a] [%]	B[b] [%]	Flow [µL/min]
0.00	99	1	250
1.00	99	1	500
2.70	0	100	500
3.70	0	100	500
3.90	99	1	250
4.90	99	1	250

[a] 0.1% ($^{v}/_{v}$) ACN, 0.05% ($^{v}/_{v}$) HCOOH in water

[b] MeOH

Table S11 Solvent settings used for Method 1C

Time [min]	A[a] [%]	B[b] [%]	Flow [µL/min]
0.00	90	10	300
1.00	90	10	300
5.00	18	82	300
5.05	0	100	300
6.00	0	100	300
6.05	90	10	400
7.00	90	10	400

[a] 0.1% ($^{v}/_{v}$) NH_4OH (>25% NH_3 in water) in water

[b] MeOH

Table S12 Solvent settings used for Method 1D

Time [min]	A[a] [%]	B[b] [%]	Flow [µL/min]
0.00	20	80	1000
9.50	40	60	1000
10.00	95	5	1000
12.00	95	5	1000
12.50	20	80	1000
18.00	20	80	1000

[a] 0.1% ($^v/_v$) ACN in water

[b] ACN

Table S13 Solvent settings used for Method 2A

Time [min]	A[a] [%]	B[b] [%]	Flow [µL/min]
0.00	95	5	400
0.50	95	5	400
0.60	50	50	400
2.50	0	100	400
3.50	0	100	400
3.55	95	5	400
4.50	95	5	400

[a] 0.1% ($^{v}/_{v}$) ACN, 0.05% ($^{v}/_{v}$) HCOOH in water

[b] MeOH

Table S14 Solvent settings used for Method 2B

Time [min]	A[a] [%]	B[b] [%]	Flow [µL/min]
0.00	90	10	500
0.50	90	10	500
0.65	61	39	500
3.00	59	41	500
3.10	50	50	500
8.50	30	70	500
9.00	0	100	500
10.00	0	100	500
10.05	90	10	500
11.00	90	10	500

[a] 0.1% ($^{v}/_{v}$) ACN, 0.05% ($^{v}/_{v}$) HCOOH in water

[b] MeOH

Table S15 Solvent settings used for Method 3

Time [min]	A[a] [%]	B[b] [%]	Flow [µL/min]
0.00	95	5	500
0.50	95	5	500
0.70	85	15	500
3.50	75	25	500
6.50	30	70	500
6.70	0	100	500
7.70	0	100	500
7.80	95	5	500
8.80	95	5	500

[a] 0.1% ($^v/_v$) ACN, 0.05% ($^v/_v$) HCOOH in water

[b] MeOH

Table S16 Validation parameter for Method 1A

Analyte[a]	LOD_i[b] [fmol]	LOQ_i[b] [fmol]	Linear[b] [fmol]	Slope	Matrix effect[c] [%]	LOQ_m [nmol]
Ala	19	68	≤ 625	1.4×10^6		3.7
$^{13}C_3$, $^{15}N_1$-Ala					79	
Arg	7.6	27	≤ 2,500	2.6×10^6		1.8
$^{13}C_6$, $^{15}N_4$-Arg					63	
Asn	28	100	≤ 2,500	9.0×10^5		5.2
$^{13}C_4$, $^{15}N_n$-Asx_{Asn}[d]					83	
Asp	8.4	30	≤ 1,250	1.1×10^6		1.5
$^{13}C_4$, $^{15}N_n$-Asx_{Asp}[d]					83	
Caffeoylputresince	15	57	≤ 995	4.9×10^6	107	2.3
Chlorogenic acid	18[e]	68[e]	≤ 706	6.1×10^6	281	1.0
Citric acid	406[e]	1330[e]	≤ 2,603	1.8×10^4	184	31
Butenedioic acid (fumaric acid & maleic acid)	55	194	≤ 4,308[f]	9.9×10^5	97	8.6
Gln	58	202	≤ 3,421[f]	1.6×10^6		11
$^{13}C_5$, $^{15}N_n$-Glx_{Gln}[d]					78	
Glu	2.7	9.5	≤ 1,250	2.4×10^6		0.52
$^{13}C_5$, $^{15}N_n$-Glx_{Glu}[d]					78	
His	6.0	21	≤ 2,500	3.2×10^6		1.7
$^{13}C_6$, $^{15}N_3$-His					54	
Ile	12	45	≤ 5,000[f]	5.7×10^6		0.22
$^{13}C_6$, $^{15}N_1$-Ile					86	
Leu	11	39	≤ 5,000[f]	6.4×10^6		0.22
$^{13}C_6$, $^{15}N_1$-Leu					75	
Lys	18	62	≤ 2,500	2.9×10^6		4.2
$^{13}C_6$, $^{15}N_2$-Lys					64	
Phe	1.2	4.3	≤ 5,000[f]	1.0×10^7		0.17
$^{13}C_9$, $^{15}N_1$-Phe					106	
Pro	6.9	24	≤ 5,000[f]	5.0×10^6		0.99

$^{13}C_5$, $^{15}N_1$-Pro					104	
Rutin	2.3	7.8	≤ 819[f]	5.4×10^5	113	0.30
Ser	9.7	34	≤ 2,500	1.3×10^6		1.9
$^{13}C_3$, $^{15}N_1$-Ser					75	
Thr	2.9	10	≤ 2,500	1.3×10^6		0.56
$^{13}C_4$, $^{15}N_1$-Thr					79	
Trp	2.1	7.9	≤ 5,000[f]	2.7×10^6	119	0.28
Tyr	0.5	2.0	≤ 5,000[f]	1.8×10^6		0.10
$^{13}C_9$, $^{15}N_1$-Tyr					89	
Tyramine	53	187	≤ 3,645[f]	2.0×10^6	85	9.4
Val	4.7	16	≤ 5,000[f]	4.7×10^6		0.59
$^{13}C_5$, $^{15}N_1$-Val					118	

LOD_i: instrument limit of detection

LOQ_i: instrument limit of quantification

LOQ_m: method limit of quantification (per sample)

[a] Compounds that are expected to have the same properties are depicted in grey

[b] Amount on column

[c] For 1 µL of 50 times diluted Fraction 1

[d] Asx and Glx handled as Asn, Asp, Glu or Gln equivalents, respectively

[e] Calculated based on only 5 (citric acid) or 6 (chlorogenic acid) dilution steps

[f] Not tested until loss of linearity (linear range potentially larger)

Table S17 Validation parameter for Method 1B

Analyte[a]	LOD$_i$[b] [fmol]	LOQ$_i$[b] [fmol]	Linear[b] [fmol]	Slope	Matrix effect[c] [%]	LOQ$_m$ [nmol]
α-Ketoglutaric acid	71[d]	256[d]	≤ 1711	1.1×10^5	71	1.5
Cystine	3.4	12	≤ 2500[e]	7.7×10^5	33	0.15
Glucuronic acid	63	223	≤ 1288	5.4×10^4	75	1.3
Gly	566[d]	1884[d]	≤ 5000[e]	6.7×10^4		20
$^{13}C_2$,$^{15}N_1$-Gly					41	
Met	6.3	22	≤ 5000[e]	1.7×10^6		0.073
$^{13}C_5$,$^{15}N_1$-Met					129	
Ornithine	9.5	34	≤ 3783[e]	1.6×10^6	36	0.40
Quercetin	18[d]	66[d]	≤ 1478[e]	2.3×10^5	105	0.27
Shikimic acid	37	133	≤ 2871[e]	6.2×10^4	20	2.8
Succinic acid	52	183	≤ 4234[e]	1.0×10^6	94	0.83
Tryptamine	1.8[d]	6.3[d]	≤ 3121[e]	1.0×10^7	108	0.25
Uracil	6.4	23	≤ 4461[e]	3.1×10^5	68	0.14
Uric acid	100[d]	363[d]	≤ 2974[e]	2.0×10^4	83	1.9
Tyr[f]				1.8×10^6		
$^{13}C_9$,$^{15}N_1$-Tyr					79	

LOD$_i$: instrument limit of detection

LOQ$_i$: instrument limit of quantification

LOQ$_m$: method limit of quantification (per sample)

[a] Compounds that are expected to have the same properties are depicted in grey

[b] Amount on column

[c] For 10 µL of 50 times diluted Fraction 1

[d] Calculated based on only 5 (Gly, α-ketoglutaric acid and quercetin) or 6 (tryptamine and uric acid) dilution steps

[e] Not tested until loss of linearity (linear range potentially larger)

[f] Usually not included in the method (just for method evaluation of $^{13}C_9$, $^{15}N_1$-Phe)

Table S18 Validation parameter for Method 1C

Analyte[a]	LOD_i[b] [fmol]	LOQ_i[b] [fmol]	Linear[b] [fmol]	Slope	Matrix effect[c] [%]	LOQ_m [pmol]
Anabasine	25	91	≤ 3082[d]	1.3×10^6	131	2969
Anatabine	0.79	2.9	≤ 3121[d]	1.2×10^7	425	29
Cotinine	0.10	0.39	≤ 709[d]	4.7×10^7	107	16
Niacin	0.37	1.4	≤ 4061[d]	1.6×10^6	73	81
D_3-Nicotine[e]	17	58	≤ 3026[d]	4.3×10^6	418	593
Nicotine						
Nornicotine	3.6[f]	13[f]	≤ 3374[d]	1.3×10^7	623	90
Scopolamine	0.046	0.17	≤ 1141[d]	4.2×10^7	252	2.9
Phe[e]				6.9×10^6		
$^{13}C_9$, $^{15}N_1$-Phe					97	

LOD_i: instrument limit of detection

LOQ_i: instrument limit of quantification

LOQ_m: method limit of quantification (per sample)

[a] Compounds that are expected to have the same properties are depicted in grey

[b] Amount on column

[c] For 1 µL of 50 times diluted Fraction 1

[d] Not tested until loss of linearity (linear range potentially larger)

[e] Usually not included in the method (just for method evaluation of nicotine and $^{13}C_9$, $^{15}N_1$-Phe, respectively)

[f] Calculated based on only 6 dilution steps

Table S19 Validation parameter for Method 1D

Analyte[a]	LOD_i[b] [fmol]	LOQ_i[b] [fmol]	Linear[b] [fmol]	Slope	Matrix effect[c] [%]	LOQ_m [nmol]
Cellubiose	41	149	≤ 2921[d]	1.4×10^5	103	62
Fructose	97	347	≤ 5551[d]	1.2×10^6	99	149
Glucose	87	313	≤ 5551[d]	8.8×10^5	99	135
Rhamnose	95	342	≤ 6092[d]	6.0×10^5	96	152
Sucrose	67	245	≤ 2921[d]	2.7×10^5	108	97
Sorbitol	71	260	≤ 5488[d]	8.6×10^5	96	115

LOD_i: instrument limit of detection

LOQ_i: instrument limit of quantification

LOQ_m: method limit of quantification (per sample)

[a] Compounds that are expected to have the same properties are depicted in grey

[b] Amount on column

[c] For 1 µL of 500 times diluted Fraction 1

[d] Not tested until loss of linearity (linear range potentially larger)

Table S20 Validation parameter for Method 2A

Analyte[a]	LOD_i[b] [fmol]	LOQ_i[b] [fmol]	Linear[b] [fmol]	Slope	Recovery rate[c] [%]	Matrix effect[d] [%]	LOQ_m [fmol]
JA-Met	0.20	0.74	≤ 732	3.8 x 10^6	51	133	1173
JA-Phe	0.12	0.44	≤ 699	7.8 x 10^6	39	100	1223
JA-Val	0.18	0.65	≤ 808	1.1 x 10^7	72	119	826
D_4-ABA	0.21	0.78	≤ 924	5.4 x 10^6	98	94	922
ABA							
D_6-JA	0.17	0.61	≤ 1156	3.5 x 10^6	83	97	828
JA							
D_6-JA-Ile	0.18	0.67	≤ 759	8.2 x 10^6	62	92	1275
JA-Ile							
D_6-SA	0.20	0.75	≤ 1759	2.3 x 10^7	86	111	848
SA							

LOD_i: instrument limit of detection

LOQ_i: instrument limit of quantification

LOQ_m: method limit of quantification (per sample)

[a] Compounds that are expected to have the same properties are depicted in grey

[b] Amount on column

[c] Recovery rate for the purification procedure, including all solid phase extraction and evaporation steps

[d] For 1 µL of Fraction 2

Table S21 Validation parameter for Method 2B

Analyte[a]	LOD_i[b] [fmol]	LOQ_i[b] [fmol]	Linear[b] [fmol]	Slope	Recovery rate[c] [%]	Matrix effect[d] [%]	LOQ_m [fmol]
Caffeic acid	28	102	≤ 2775	1.6×10^6	90	20	6501
Cinnamic acid	247[e]	850[e]	≤ 3375[f]	8.6×10^4	38	98	26080
Coniferyl aldehyde	8.2	31	≤ 2806[f]	7.3×10^5	63	69	764
para-Coumaric acid	3.7	14	≤ 3046[f]	2.6×10^6	88	50	355
meta-Coumaric acid	5.5	20	≤ 3046[f]	1.7×10^6	98	56	424
ortho-Coumaric acid	9.1	33	≤ 3046[f]	1.8×10^6	110	54	635
Ferulic acid	13	48	≤ 2575[f]	3.9×10^5	114	49	979
Fraxetin	5.8	21	≤ 2402[f]	7.8×10^5	92	50	516
GA_1	10	36	≤ 1435[f]	2.0×10^5	90	49	939
GA_4	2.1	6.6	≤ 1053[f]	4.4×10^5	69	78	140
GA_5	39[e]	138[e]	≤ 757[f]	6.1×10^4	89	111	1602
GA_7	0.47	1.7	≤ 545[f]	3.1×10^6	76	70	36
GA_8	17[e]	63[e]	≤ 686[f]	4.2×10^4	63	32	3584
GA_9	22[e]	81[e]	≤ 790[f]	3.6×10^4	14	79	8421
GA_{12}	9.4	34	≤ 752[f]	5.6×10^4	14	20	13922
GA_{12ald}					0.64		
GA_{13}	29	104	≤ 661[f]	1.8×10^5	92	126	1021
GA_{20}	24[e]	86[e]	≤ 752[f]	2.7×10^4	87	106	1060
GA_{24}	40[e]	141[e]	≤ 722[f]	2.9×10^4	49	99	3322
GA_{29}	59[e]	199[e]	≤ 718[f]	2.1×10^4	77	12	24651
GA_{44}	29[e]	104[e]	≤ 722[f]	1.6×10^4	83	113	1267
GA_{51}	11	40	≤ 752[f]	2.4×10^5	82	109	514
IA-Ala	3.1	11	≤ 2030[f]	1.9×10^6	107	84	143
IAM	0.90	3.3	≤ 1435	2.7×10^6	82	69	67
IBA	2.7	10	≤ 2460[f]	1.4×10^6	49	78	302
Scopoletine	7.9	29	≤ 2602[f]	2.2×10^5	77	69	631
Sinapic acid	101[e]	358[e]	≤ 2230[f]	6.8×10^5	102	42	9641
Sinapyl aldehyd[g]					68	13	

4-MU	36	131	≤ 2838[f]	2.2×10^5	96	65	2393
D_5-IAA	13	49	≤ 499[f]	6.0×10^5	88	26	2425
IAA	3.8	14					679
D_2-GA_3[h]	5.0	19	≤ 1435[f]	3.8×10^5	83	44	583
GA_3							

LOD_i: instrument limit of detection

LOQ_i: instrument limit of quantification

LOQ_m: method limit of quantification (per sample)

4-MU: 4-methylumbelliferone

[a] Compounds that are expected to have the same properties are depicted in grey

[b] Amount on column

[c] Recovery rate for the purification procedure, including all solid phase extraction and evaporation steps, excluding the removal of an aliquot for the analysis by Method 2A

[d] For 5 µL of 20 times concentrated Fraction 2

[e] Calculated based on only 5 (GA_{24} and GA_{29}) or 6 (cinnamic acid, sinapic acid, GA_5, GA_8, GA_9, GA_{20} and GA_{44}) dilution steps

[f] Not tested until loss of linearity (linear range potentially larger)

[g] Tested with standard of unknown concentration

[h] Usually not included in the method (just for method evaluation of GA_3)

Table S22 Validation parameter for Method 3

Analyte[a]	LOD_i[b] [fmol]	LOQ_i[b] [fmol]	Linear[b] [fmol]	Slope	Recovery rate[c] [%]	Matrix effect[d] [%]	LOQ_m [fmol]
BAP	2.1	7.2	≤ 1110	8.3 x 10^6	4	77	4621
DHZ*7*G	0.38	1.4	≤ 1304[e]	1.2 x 10^6	80	55	58
DHZ*9*G	0.35	1.3	≤ 1304[e]	2.7 x 10^6	88	60	45
DHZ*O*G	0.27	0.99	≤ 1304[e]	1.4 x 10^6	67	66	43
DHZR*O*G	0.39	1.4	≤ 970[e]	2.0 x 10^6	84	63	49
IP*9*G	0.41	1.5	≤ 684	4.7 x 10^6	66	77	55
D_3-DHZ	0.15	0.55	≤ 1115[e]	9.1 x 10^6	33	40	79
DHZ							
D_3-DHZR	0.11	0.39	≤ 140[e]	2.1 x 10^7	70	42	25
DHZR							
D_6-IP	0.076	0.27	≤ 478[e]	2.6 x 10^7	3	60	264
IP							
D_6-IPR	0.032	0.11	≤ 293[e]	2.7 x 10^7	15	76	19
IPR							
D_5-*t*Z	0.18	0.66	≤ 2230[e]	6.7 x 10^6	33	50	78
*t*Z							
*c*Z							
D_5-*t*ZR	0.21	0.74	≤ 281[e]	7.0 x 10^6	75	59	32
*t*ZR							
*c*ZR							
D_5-*t*Z*7*G	0.12	0.41	≤ 2588[e]	7.8 x 10^6	85	46	20
*t*Z*7*G							
*c*Z*7*G							
D_5-*t*Z*9*G	0.20	0.72	≤ 2588[e]	5.7 x 10^6	92	64	23
*t*Z*9*G							
*c*Z*9*G							
D_5-*t*Z*O*G	0.25	0.87	≤ 5176[e]	2.2 x 10^6	71	57	41

*t*Z*O*G							
*c*Z*O*G							
D_5-*t*ZR*O*G	0.42	1.5	≤ 7714[e]	2.2×10^6	85	62	54
*t*ZR*O*G							
*c*ZR*O*G							

LOD_i: instrument limit of detection

LOQ_i: instrument limit of quantification

LOQ_m: method limit of quantification (per sample)

[a] Compounds that are expected to have the same properties are depicted in grey

[b] Amount on column

[c] Recovery rate for the purification procedure, including all solid phase extraction and evaporation steps

[d] For 3 µL of 20 times concentrated Fraction 3

[e] Not tested until loss of linearity (linear range potentially larger)

Table S23 Re-analysis of selected samples[a] after a prolonged storage time[b]

	Compound abundance relative to first analysis [%]	SE		Compound abundance relative to first analysis [%]	SE
**Method 1A**			**Method 1B**		
Ala	103	5.6	Gly	109	1.9
Arg	110	13	Met	101	1.2
Asn	106	2.2	L-Ornithin	137	26
Asp	94	2.2	Tryptamine	191	32
Glu	108	5.3	a-Ketoglutaric acid	71	4
Gln	126	9.1	Glucoronic acid	96	17
His	88	8.7	Succinic acid	116	3.0
Ile	112	15	Uric acid	79	6.9
Leu	130	29	Quercetin	174	81
Lys	160	33	Shikimic acid	223	43
Phe	106	8.3			
Pro	108	9.1			
Ser	105	3.4	**Method 1C**		
Thr	102	2.6	Nicotine	101	3.4
Trp	80	7.0	Nornicotine	117	2.1
Tyramine	196	11	Anatabine	122	4.3
Tyr	114	11			
Val	106	7.6			
Caffeoylputrescine	162	18	**Method 1D**		
Chlorogenic acid	171	35	Glucose	106	14
Rutin	166	18	Fructose	100	7.4
Butenedioic acid	98	10	Succrose	112	13
Malic acid	98	17			

SE: Standard error

[a] Five biological replicates of leave samples after 1 hour simulated herbivory
[b] Between the analyzes samples were kept for > 1 d at 10°C, > 20 weeks at -20°C and faced additional melting-freezing cycles

Table S24 Data from two example experiments examining metabolic responses of leaves to herbivory and during seed development

Analyte	Method	Unit	Experiment 1 (herbivory response)						Experiment 2 (seed development)					
			C		W+OS 1h		W+OS 2d		Flower base[a]		Green capsule		Seeds	
			Average	SE	Average	SE	Average	SE	Average	SE	Average	SE	Average	SE
Ala	1A	nmol * g FW^{-1}	269	19	566	22	433	17	568	52	398	61	220	15
Arg	1A	nmol * g FW^{-1}	64	8.9	82	5.7	29	1.4	117	15	114	18	154	7.0
Asn	1A	nmol * g FW^{-1}	206	21	238	10	118	6.7	1606	179	917	141	1512	137
Asp	1A	nmol * g FW^{-1}	1232	56	970	34	1057	22	2970	265	1572	119	529	24
Gln	1A	nmol * g FW^{-1}	3737	488	4504	293	2278	136	51278	4252	5995	1056	653	54
Glu	1A	nmol * g FW^{-1}	3627	153	2542	67	1980	125	6681	362	2625	190	2419	82
His	1A	nmol * g FW^{-1}	42	3.0	48	4.6	32	0.76	2324	338	176	10	135	11
Ile	1A	nmol * g FW^{-1}	28	2.0	39	2.3	25	1.2	197	34	123	11	104	3.7
Leu	1A	nmol * g FW^{-1}	56	3.2	98	4.2	55	2.3	478	69	171	7.4	263	14
Lys	1A	nmol * g FW^{-1}	105	8.2	95	3.7	88	6.8	231	40	141	11	205	15
Phe	1A	nmol * g FW^{-1}	74	5.6	121	4.4	85	1.8	1070	204	56	6.4	203	7.2
Pro	1A	nmol * g FW^{-1}	75	7.0	75	3.9	48	2.4	835	140	383	88	2579	78
Ser	1A	nmol * g FW^{-1}	709	52	728	43	539	21	2094	194	807	88	888	50
Thr	1A	nmol * g FW^{-1}	349	23	428	14	321	10	2288	156	642	127	292	9.1
Trp	1A	nmol * g FW^{-1}	12	1.8	21	2.4	12	1.1	2672	350	413	58	806	95
Tyr	1A	nmol * g FW^{-1}	14	1.2	22	1.6	15	0.76	134	20	57	14	103	3.4
Tyramine	1A	nmol * g FW^{-1}	17	1.4	26	3.6	576	32	626	61	443	40	<LOD	
Val	1A	nmol * g FW^{-1}	75	5.4	117	4.9	71	2.1	555	67	177	15	439	6.6
Cystine	1B	nmol * g FW^{-1}	<LOD		<LOD		<LOD		<LOD		<LOD		4.4	0.44
Gly	1B	nmol * g FW^{-1}	323	41	618	36	184	7	427	40	183	23	282	26
Met	1B	nmol * g FW^{-1}	54	2.9	54	3.1	28	1.0	72	9.6	73	8.2	44	3.5
L-Ornithin	1B	nmol * g FW^{-1}	8.9	1.7	14	2.1	6.8	1.3	74	5.9	25	3.2	37	5.2
Tryptamine	1B	nmol * g FW^{-1}	0.069	0.0059	0.064	0.011	0.17	0.016	0.87	0.14	0.66	0.22	0.31	0.037

Citric acid	1A	nmol * g FW^{-1}	215	149	509	351	<LOD		36[b]	36[b]	769[b]	230[b]	45[b]	24[b]
Butenedioic acid (fumaric acid & maleic acid)	1A	nmol * g FW^{-1}	315	23	256	26	231	21	<LOD		<LOD		<LOD	
Malic acid	1A	Rel. quant.	9083	681	10589	354	13706	1143	24762	4803	10502	606	198	39
a-Ketoglutaric acid	1B	nmol * g FW^{-1}	484	39	486	30	519	36	1076	181	699	99	62	14
Glucoronic acid	1B	nmol * g FW^{-1}	19	3.7	19	2.5	20	3.1	194	27	126	28	25	2.2
Succinic acid	1B	nmol * g FW^{-1}	76	3.3	251	16	32	1.5	151	13	508	42	138	10
Uric acid	1B	nmol * g FW^{-1}	26	7.6	49	26	23	3.8	6652	1017	1032	62	128	40
Niacin	1C	nmol * g FW^{-1}	<LOD		<LOD		<LOD		<LOD		0.39	0.19	80	3.4
Glucose	1D	nmol * g FW^{-1}	29509	1570	31340	3161	23496	2361	30752	5775	30029	13042	554	110
Fructose	1D	nmol * g FW^{-1}	18430	1174	20918	1469	15802	1715	22741	2556	18569	6846	609	110
Succrose	1D	nmol * g FW^{-1}	7752	325	10697	687	11661	673	51782	7206	16210	2206	39925	4055
Caffeoylputrescine	1A	nmol * g FW^{-1}	90	14	135	40	2349	183	1270	150	196	49	<LOD	
Chlorogenic acid	1A	nmol * g FW^{-1}	881	85	971	73	1047	56	4365	792	9634[b]	803[b]	1117	199
Rutin	1A	nmol * g FW^{-1}	1102	102	1436	128	1332	131	1819	242	275	47	341	54
Quercetin	1B	nmol * g FW^{-1}	0.39	0.13	0.35	0.069	0.39	0.061	13	1.3	4.6	1.0	<LOD	
Shikimic	1B	nmol * g FW^{-1}	134	26	137	15	102	14	437	76	366	38	17	3.0
Nicotine	1C	nmol * g FW^{-1}	957	151	856	112	1467	216	11472	3629	5266	605	59	6.6
Nornicotine	1C	nmol * g FW^{-1}	0.58	0.085	0.56	0.079	0.89	0.15	31	8.2	9.4	1.6	<LOD	
Cotinine	1C	nmol * g FW^{-1}	<LOD		<LOD		0.020	0.0048	<LOD		0.014	0.0039	<LOD	
Anatabine	1C	nmol * g FW^{-1}	12	2.3	9.9	1.5	15	3.2	52	17	14	1.7	<LOD	
Cinnamic acid	2B	pmol * g FW^{-1}	95	16	439	27	138	12	<LOD		209	35	128	7.7
para-Coumaric acid	2B	pmol * g FW^{-1}	229	23	326	13	160	13	121	16	295	97	319	30
Caffeic acid	2B	pmol * g FW^{-1}	527	31	980	65	943	49	1363	161	7041	1719	1189	62
Ferulic acid	2B	pmol * g FW^{-1}	27	2.1	67	3.7	22	1.5	275	29	1174	174	997	41
Scopoletine	2B	pmol * g FW^{-1}	1.2	0.27	6.8	0.62	6.4	0.49	8.3	3.7	100	68	501	52
Scopoline	2B	Rel. quant.	66	3.5	72	4.9	333	19	445	52	131	28	399	61

Coniferyl aldehyd	2B	pmol * g FW^{-1}	13	3.3	19	2.5	12	2.0	108	6.4	253	34	3670	437
Sinapic acid	2B	pmol * g FW^{-1}	298	14	393	19	289	12	898	89	496	69	66	10
Sinapyl aldehyd	2B	Rel. quant.	15	1.1	17	0.80	19	1.7	381	25	91	20	9.9	1.4
ABA	2A	pmol * g FW^{-1}	251	18	533	22	408	26	4165	256	990	158	359	58
SA	2A	pmol * g FW^{-1}	251	17	476	55	468	38	1377	66	269	24	580	54
JA	2A	pmol * g FW^{-1}	6.2	2.7	13873	1202	16	4.5	93	32	3.7	2.4	1147	30
JA-Ile	2A	pmol * g FW^{-1}	<LOD		477	55	<LOD		<LOD		<LOD		1245	79
JA-Met	2A	pmol * g FW^{-1}	<LOD		17	1.2	<LOD		<LOD		<LOD		<LOD	
JA-Val	2A	pmol * g FW^{-1}	<LOD		53	1.8	<LOD		<LOD		<LOD		26	3.7
OH-JA	2A	Rel. quant.	88	37	6993	734	396	129	447	44	201	59	4884	350
OH-JA-Ile	2A	Rel. quant.	5.2	1.4	2851	136	2.1	0.58	54	8.8	33	5.1	85	9.1
COOH-JA-Ile	2A	Rel. quant.	<LOD		1341	131	7.1	0.46	14	3.9	26	7.7	22	4.7
IAA	2B	pmol * g FW^{-1}	69	4.9	63	1.9	37	1.1	48	5.8	272	60	67	6.1
GA_1	2B	pmol * g FW^{-1}	0.84	0.58	<LOD		<LOD		<LOD		1.1	1.1	<LOD	
GA_3	2B	pmol * g FW^{-1}	2.3	0.48	0.83	0.17	2.3	0.56	6.3	1.2	6.5	3.1	27	10
GA_8	2B	pmol * g FW^{-1}	80	5.2	107	5.6	67	6.9	40	3.9	114	14	<LOD	
GA_{20}	2B	pmol * g FW^{-1}	29	4.0	39	5.0	2.6	1.3	<LOD		<LOD		<LOD	
GA_{29}	2B	pmol * g FW^{-1}	234	17	283	13	276	21	243	77	<LOD		19	19
GA_{51}	2B	pmol * g FW^{-1}	<LOD		<LOD		<LOD		<LOD		2.3	0.84	<LOD	
IP	3	pmol * g FW^{-1}	0.061	0.0050	0.050	0.0039	0.049	0.0038	0.61	0.044	0.12	0.0061	<LOD	
IPR	3	pmol * g FW^{-1}	0.90	0.048	1.4	0.11	0.54	0.039	15	2.1	0.48	0.084	<LOD	
*t*Z	3	pmol * g FW^{-1}	1.2	0.13	0.91	0.047	1.1	0.077	0.97	0.094	0.36	0.076	<LOD	
*t*ZR	3	pmol * g FW^{-1}	2.0	0.25	0.85	0.083	0.64	0.036	29	3.4	8.5	2.0	1.4	0.076
*c*Z	3	pmol * g FW^{-1}	0.069	0.010	0.072	0.015	0.15	0.027	<LOD		0.44	0.071	0.52	0.073
*c*ZR	3	pmol * g FW^{-1}	0.30	0.016	1.5	0.13	0.28	0.052	8.2	0.99	7.9	1.4	21	0.82
DHZ	3	pmol * g FW^{-1}	0.32	0.034	0.42	0.029	0.34	0.027	<LOD		0.22	0.027	<LOD	
DHZR	3	pmol * g FW^{-1}	0.26	0.042	0.25	0.026	0.076	0.011	0.79	0.13	2.9	0.34	2.5	0.12
*c*ZOG	3	pmol * g FW^{-1}	1.1	0.081	1.1	0.080	3.2	0.13	5.8	0.59	2.8	0.17	<LOD	
DHZOG	3	pmol * g FW^{-1}	0.42	0.11	0.38	0.082	0.31	0.061	<LOD		1.2	0.26	3.0	2.0

*t*ZR*O*G	3	pmol * g FW^{-1}	1.3	0.079	1.2	0.089	1.1	0.061	76	14	16	2.5	<LOD	
*c*ZR*O*G	3	pmol * g FW^{-1}	2.8	0.18	3.1	0.34	5.9	0.29	73	2.7	22	2.1	0.64	0.039
DHZR*O*G	3	pmol * g FW^{-1}	2.7	0.31	2.8	0.37	3.3	0.28	16	0.62	9.5	1.4	<LOD	
*t*Z*7*G	3	pmol * g FW^{-1}	5.0	0.19	4.8	0.13	6.8	0.21	6.6	0.52	7.1	0.91	0.19	0.035
*c*Z*7*G	3	pmol * g FW^{-1}	2.4	0.25	1.9	0.12	5.8	0.29	19	1.4	15	1.1	14	0.36
DHZ*7*G	3	pmol * g FW^{-1}	30	1.6	31	1.1	30	1.0	22	3.2	40	6.5	14	0.75
DHZ*9*G	3	pmol * g FW^{-1}	0.083	0.049	0.13	0.063	0.037	0.025	<LOD		0.068	0.068	<LOD	

SE: Standard error (experiment 1, n=10; experiment 2, n≥6)

Rel. quant.: relative quantification based on the internal standards mentioned in Table S2-S8; without response factor

LOD: limit of detection

FW: fresh weight

[a] Flower (already opened and likely pollinated) with removed corolla, stamen, style and pedicel

[b] Relative quantification (based on the internal standards mentioned in Table S2-S8; without response factor) for citric acid in Experiment 2 and chlorogeneic acid for Green capsules

Table S25 Response factors use for the analysis of flower and seed extracts.

		Response factor[a]		
Analyte	Standard	Flower base[b]	Green capsule	Seeds
Caffeoylputresine	$^{13}C_9$, $^{15}N_1$-Tyr	0.30	0.34	0.38
Chlorogenic acid	$^{13}C_9$, $^{15}N_1$-Tyr	8.0	-	3.5
Citric acid	$^{13}C_9$, $^{15}N_1$-Tyr	-	-	-
Butenedioic acid (fumaric acid & maleic acid)	$^{13}C_9$, $^{15}N_1$-Tyr	1.5	1.3	1.8
Rutin	$^{13}C_9$, $^{15}N_1$-Tyr	2.3	2.2	3.8
Trp	$^{13}C_9$, $^{15}N_1$-Phe	4.3	3.3	4.2
Tyramine	$^{13}C_9$, $^{15}N_1$-Tyr	0.86	0.91	0.87
α-Ketoglutaric acid	$^{13}C_9$, $^{15}N_1$-Tyr	17	19	19
Cystine	$^{13}C_9$, $^{15}N_1$-Tyr	5.7	7.3	5.6
Glucuronic acid	$^{13}C_9$, $^{15}N_1$-Tyr	50	33	51
Ornithine	$^{13}C_6$, $^{15}N_2$-Tyr	2.9	3.6	2.4
Quercetin	$^{13}C_9$, $^{15}N_1$-Tyr	5.6	5.3	6.0
Shikimic acid	$^{13}C_9$, $^{15}N_1$-Tyr	43	91	50
Succinic acid	$^{13}C_9$, $^{15}N_1$-Tyr	1.4	2.2	1.6
Tryptamine	$^{13}C_9$, $^{15}N_1$-Tyr	0.15	0.14	0.19
Uracil	$^{13}C_9$, $^{15}N_1$-Tyr	6.3	8.6	6.8
Uric acid	$^{13}C_9$, $^{15}N_1$-Tyr	149	250	106
Anabasine	$^{13}C_9$, $^{15}N_1$-Phe	3.6	2.4	4.3
Anatabine	$^{13}C_9$, $^{15}N_1$-Phe	0.36	0.20	0.46
Cotinine	$^{13}C_9$, $^{15}N_1$-Phe	0.11	0.056	0.13
Niacin	$^{13}C_9$, $^{15}N_1$-Phe	4.8	5.0	6.5
Nicotine	$^{13}C_9$, $^{15}N_1$-Phe	1.1	0.99	1.2
Nornicotine	$^{13}C_9$, $^{15}N_1$-Phe	0.33	0.18	0.37
Scopolamine	$^{13}C_9$, $^{15}N_1$-Phe	0.12	0.078	0.14

Cellubiose	Sorbitol	6.1	5.1	5.5
Fructose	Sorbitol	0.71	0.64	0.68
Glucose	Sorbitol	0.95	0.79	0.90
Rhamnose	Sorbitol	1.4	1.1	1.3
Sucrose	Sorbitol	2.9	1.9	2.2
JA-Met	D_6-JA-Ile	2.4	1.9	2.4
JA-Phe	D_6-JA-Ile	2.1	2.5	1.7
JA-Val	D_6-JA-Ile	0.59	0.47	0.61
Caffeic acid	4-MU	0.34	1.2	0.23
Cinnamic acid	4-MU	5.2	6.4	6.2
Coniferyl aldehyde	4-MU	0.34	0.37	0.41
para-Coumaric acid	4-MU	0.14	0.25	0.17
meta-Coumaric acid	4-MU	0.15	0.21	0.15
ortho-Coumaric acid	4-MU	0.12	0.15	0.13
Ferulic acid	4-MU	0.58	0.93	0.60
Fraxetin	4-MU	0.35	1.2	0.37
GA_1	4-MU	1.3	2.3	1.4
GA_3	4-MU	0.83	1.9	1.4
GA_4	4-MU	0.60	1.0	2.5
GA_5	4-MU	3.4	5.6	4.0
GA_7	4-MU	0.084	0.11	0.17
GA_8	4-MU	16	88	18
GA_9	4-MU	41	63	201
GA_{12}	4-MU	20	48	30
GA_{13}	4-MU	1.0	0.91	1.3
GA_{20}	4-MU	7.2	9.7	10
GA_{24}	4-MU	13	19	56
GA_{29}	4-MU	40	212	25
GA_{44}	4-MU	15	19	18

GA_{51}	4-MU	0.90	1.1	1.1
IA-Ala	D_5-IAA	0.51	0.45	0.73
IAM	D_5-IAA	0.57	0.70	0.72
IBA	D_5-IAA	2.1	3.4	3.2
Scopoletine	4-MU	1.4	1.5	1.6
Sinapic acid	4-MU	0.48	1.1	0.66
BAP	D_6-IP	2.5	2.2	2.7
DHZ*7*G	D_5-*t*Z*9*G	6.0	6.0	5.1
DHZ*9*G	D_5-*t*Z*9*G	2.4	2.4	2.2
DHZ*O*G	D_5-*t*Z*9*G	6.4	6.2	6.6
DHZR*O*G	D_3-*t*ZR*O*G	1.1	1.1	1.1
IP*9*G	D_6-IP	0.20	0.19	0.22

4-MU: 4-methylumbelliferone

[a] Matrix and recovery corrected

[b] Flower (already opened and likely pollinated) with removed corolla, stamen, style and pedicel
